# Surface-engineered extracellular vesicles for targeted delivery of therapeutic RNAs and peptides for cancer therapy

**DOI:** 10.7150/thno.68667

**Published:** 2022-04-11

**Authors:** Migara Kavishka Jayasinghe, Marco Pirisinu, Yuqi Yang, Boya Peng, Thach Tuan Pham, Chang Yu Lee, Melissa Tan, Luyen Tien Vu, Xuan TT Dang, Tin Chanh Pham, Huan Chen, Anskar Y.H. Leung, William C. Cho, Jiahai Shi, Minh TN Le

**Affiliations:** 1 Department of Pharmacology and Institute for Digital Medicine, Yong Loo Lin School of Medicine, National University of Singapore, 16 Medical Drive, Singapore.; 2 Department of Surgery, Immunology Programme, Cancer Programme and Nanomedicine Translational Programme, Yong Loo Lin School of Medicine, National University of Singapore, 1E Kent Ridge Road, Singapore.; 3 Department of Biomedical Sciences, Jockey Club College of Veterinary Medicine and Life Sciences, City University of Hong Kong, Tat Chee Avenue, Kowloon, Hong Kong.; 4 City University of Hong Kong Shenzhen Institute, Shenzhen, Guangdong, China.; 5 Queen Mary Hospital and Department of Medicine, Li Ka Shing Faculty of Medicine, The University of Hong Kong, Pok Fu Lam, Hong Kong Island, Hong Kong.; 6 Department of Clinical Oncology, Queen Elizabeth Hospital, 30 Gascoigne Rd, Kowloon, Hong Kong.; 7 Department of Biochemistry and Synthetic Biology Program, Yong Loo Lin School of Medicine, National University of Singapore, 8 Medical Drive, Singapore.

## Abstract

The advent of novel therapeutics in recent years has urged the need for a safe, non-immunogenic drug delivery vector capable of delivering therapeutic payloads specifically to diseased cells, thereby increasing therapeutic efficacy and reducing side effects. Extracellular vesicles (EVs) have garnered attention in recent years as a potentially ideal vector for drug delivery, taking into account their intrinsic ability to transfer bioactive cargo to recipient cells and their biocompatible nature. However, natural EVs are limited in their therapeutic potential and many challenges need to be overcome before engineered EVs satisfy the levels of efficiency, stability, safety and biocompatibility required for therapeutic use.

Here, we demonstrate that an enzyme-mediated surface functionalization method in combination with streptavidin-mediated conjugation results in efficient surface functionalization of EVs. Surface functionalization using the above methods permits the stable and biocompatible conjugation of peptides, single domain antibodies and monoclonal antibodies at high copy number on the EV surface. Functionalized EVs demonstrated increased accumulation in target cells expressing common cancer associated markers such as CXCR4, EGFR and EpCAM both *in vitro* and *in vivo*. The functionality of this approach was further highlighted by the ability of targeting EVs to specifically deliver therapeutic antisense oligonucleotides to a metastatic breast tumor model, resulting in increased knockdown of a targeted oncogenic microRNA and improved metastasis suppression. The method was also used to equip EVs with a bifunctional peptide that targets EVs to leukemia cells and induces apoptosis, leading to leukemia suppression. Moreover, we conducted extensive testing to verify the biocompatibility, and safety of engineered EVs for therapeutic use, suggesting that surface modified EVs can be used for repeated dose treatment with no detectable adverse effects. This modular, biocompatible method of EV engineering offers a promising avenue for the targeted delivery of a range of therapeutics while addressing some of the safety concerns associated with EV-based drug delivery.

## Introduction

Despite significant advances in drug discovery, the majority of current drugs are limited in their therapeutic efficacy and/or safety due to poor biodistribution, difficulty in penetrating cellular barriers, the onset of adverse side effects and complications brought upon by the need for repeated dosages. Drug delivery vectors have garnered increased attention in recent years as vehicles that can efficiently shuttle therapeutic payloads to diseased cells, thereby addressing much of the aforementioned problems. This is particularly true in the case of RNA and peptide-based drugs, where drug delivery vectors could help overcome obstacles such as rapid renal clearance, degradation and poor cellular permeability. However, the majority of drug delivery vectors currently in use, including synthetic nanoparticles and viruses, bring with them their own complications in terms of safety and biocompatibility 1.

Extracellular vesicles (EVs), a class of naturally released, cell-derived, lipid bilayer enclosed nanoparticles, have been increasingly exploited as alternative drug delivery vectors, taking into account their innate ability to efficiently facilitate the transport of functional biomolecules between cells while protecting them from renal clearance and degradation 2. Yet, despite their many favorable characteristics, unmodified EVs demonstrate limited therapeutic potential owing to their non-specific pattern of uptake. In recent years, this has led to the emergence of a novel drug delivery vector in the form of engineered EVs, where EVs are artificially engineered to varying degrees via the incorporation of therapeutic molecules in conjunction with surface functionalization 3. Surface functionalization aims to improve the pharmacokinetics of EVs, actively directing EVs to desired cells via targeting moieties immobilized on the EV surface, as well as by decreasing non-specific uptake or delaying phagocytic clearance. The combination of these strategies can decrease the onset of side effects, while allowing targeting EVs to be efficiently internalized by diseased cells, bypassing cellular barriers through natural endocytic processes 4.

However, many existing methods for engineering EVs lack a balance between efficiency, biocompatibility and stability. While popular approaches based on parental cell transfection and lipid insertion have been widely utilized by multiple groups to engineer the surface of EVs, these approaches present certain limitations that hinder their successful clinical translation. Some of the major concerns associated with existing methods include the risk of mutagenic nucleic acid transfer from parental cells that are frequently transduced using viruses, the transient nature of lipid insertion and the ability of certain chemical-based methods to compromise or alter the EVs' endogenous properties 4. There are also concerns regarding the scalability and reproducibility of these approaches, given the tedious and costly nature of EV isolation, a barrier not encountered by synthetic liposomal formulations 5.

Here, we present a drug delivery platform based on red blood cell-derived EVs (RBCEVs) that are efficiently functionalized post-isolation via an enzymatic approach to facilitate antigen-specific tumor cell targeting. Given their natural origin, human RBCEVs are non-immunogenic, biocompatible and importantly, free of any oncogenic DNA/RNA 6, 7. Moreover, a homogenous population of RBCEVs can be produced at very high yield with minimal cost, ensuring the scalability of the platform 6. To facilitate targeted delivery of RBCEVs, we utilized OaAEP1-mediated ligation alone or in combination with the biotin-streptavidin system to achieve stable surface functionalization of RBCEVs. This is an advancement from our recent innovation of the enzymatic conjugation method for modifying the surface of EVs using OaAEP1 ligase 8. In the present study, we demonstrate a significant improvement in the efficiency and versatility of EV surface functionalization via the utilization of specially designed peptides and the biotin-streptavidin affinity interaction which has enabled us to increase the copy number of functional peptides and single domain antibodies (also called nanobodies) per EV. More significantly, we demonstrate the ability to stably conjugate intact monoclonal antibodies (mAb) on the EV surface with comparable efficiency, further extending the scope of EV targeting.

To display the improved efficacy and flexibility of this approach, RBCEVs were conjugated with peptides, single domain antibodies or monoclonal antibodies against CXC-chemokine receptor 4 (CXCR4), epidermal growth factor receptor (EGFR) and epithelial cell adhesion molecule (EpCAM), respectively. Engineered RBCEVs targeting these cancer-associated cell surface receptors increased the specific accumulation of encapsulated payloads in target cells. Furthermore, from a therapeutic standpoint, we establish the ability of these engineered EVs to efficiently and specifically deliver either a bifunctional apoptotic peptide targeted to CXCR4 to suppress leukemia, or an antisense oligonucleotide (ASO) to knockdown oncogenic miR-125b in EGFR-expressing breast cancer cells, resulting in decreased cell viability in both cases. Lastly, we substantiated the potential of this engineering approach for clinical translation via the treatment of leukemia xenografted mice using RBCEVs ligated with a bifunctional pro-apoptotic peptide, resulting in efficient leukemia suppression *in vivo*. Similar therapeutic effects were also observed with ASO-loaded RBCEVs targeted to an EGFR-positive metastatic breast cancer allograft. We also assessed engineered RBCEVs for potential toxicity and immunogenicity to ascertain their suitability for clinical applications. Engineered RBCEVs possessed a non-toxic, biocompatible profile similar to that of unmodified RBCEVs and showed no evidence of acute inflammatory responses upon administration. Moreover, engineered RBCEVs retained similar physicochemical characteristics as that of unmodified RBCEVs, confirming the gentle and non-destructive approach used for surface functionalization.

Hence, the surface functionalization approach utilized in this study enables the development of EV-based drug delivery vectors with improved functionality and versatility, capable of antigen-specific targeting mediated by a range of targeting molecules. Importantly, the increased functionality does not negatively impact the biocompatible profile of the engineered EVs. Targeted delivery of RNA or peptide-based drugs using surface functionalized RBCEVs can increase treatment efficacy and decrease side effects while concurrently maintaining the desired safety levels and scalability that frequently obstruct clinical translation of EV-based therapies.

## Materials and Methods

### RBCEV purification

Whole blood was obtained from patients with informed consent by the Hong Kong Red Cross in single-system blood bags (Macopharma, France). For this study, blood was obtained exclusively from O negative blood type individuals. Subsequent processing was performed following approved ethical guidelines. In short, platelet rich plasma was removed using an initial step of centrifugation (1000 × g for 8 minutes at 4 °C) followed by three washes with PBS (1000 × g for 8 minutes at 4 °C). The subsequent cell pellet was resuspended in PBS and passed through a leukodepletion filter to remove white blood cells (Nigale, China). The flowthrough containing purified red blood cells (RBCs) was collected in Nigale buffer (0.2 g/L citric acid, 1.5 g/L sodium citrate, 7.93 g/L glucose, 0.94 g/L sodium dihydrogen phosphate, 0.14 g/L adenine, 4.97 g/L sodium chloride, 14.57 g/L mannitol). This RBC suspension was diluted up to three times in PBS containing 0.1 mg/mL calcium chloride and incubated overnight with calcium ionophore (Sigma-Aldrich, USA) at a final concentration of 10 μM at 37 °C with 5% CO_2_ to induce vesiculation. The resulting RBCEVs were isolated from the RBCs as described in our previous study 6. Following isolation and purification, RBCEVs were stored in PBS supplemented with 4% trehalose as a cryoprotectant at -80 °C.

For tracking RBCEV uptake *in vitro*, EVs were labelled with carboxyfluorescein diacetate succinimidyl ester (CFSE), a cell permeant dye that is fixed inside cells and EVs by intracellular/intraluminal esterases, resulting in a well-retained fluorescent signal. For CFSE labelling, 1 mg of RBCEVs were incubated for 1 hour at 37˚C with 4 μL of 5 mM CFSE stock dissolved in DMSO to achieve a final CFSE concentration of 20 μM. Following labelling, RBCEVs were spun down at 21,000 × g. The resulting pellet was resuspended in PBS and passed through a qEV-original size exclusion chromatography (SEC) column (Izon, New Zealand). The fractions containing RBCEVs (fraction 7-9) were collected and washed twice at 21,000 × g for 20 minutes at 4 °C using centrifugation.

### RBCEV characterization

The size distribution and concentration of RBCEVs were quantified utilizing a NanoSight Tracking Analysis NS300 system (Malvern, UK) or a ZetaView® Particle Tracking Analysis instrument (Particle Metrix, Germany). Prior to analysis, RBCEVs were diluted 10,000-fold in sterile, filtered PBS. The polydispersity index of different RBCEV treatments was directly obtained using a Litesizer (Anton-Paar), at a dilution of 1000-fold in PBS. For the measurement of zeta potential, RBCEVs were diluted 10,000-fold in 10 mM HEPES buffer, the same buffer used for the dilution of the calibration beads. For general quantification of RBCEVs throughout the project, the hemoglobin (Hb) content of RBCEVs was used as an indication of RBCEV quantity. Hb content was quantified using a Nanodrop 2000 instrument (Thermo Fisher Scientific), by measuring absorbance at isosbestic points for Hb/Oxy-Hb (420, 586 nm). Nanoparticle tracking analysis (NTA) via the ZetaView® Particle Tracking Analysis instrument was used for obtaining EV concentration (and thereby EV count) for experiments requiring quantification of molecules per EV.

### Nanobody and peptide design and synthesis

Peptides were designed to contain a functional domain at the N-terminal with a flexible linker sequence separating it from the ligation motif as listed in Table S1. Peptides were synthesized using 96/102 well automated peptide synthesizers followed by purification using HPLC (GL Biochem Ltd., Shanghai, China). The nanobody sequence for EGFR variable heavy homodimers (VHH) was obtained from Roovers et al. 9 and cloned with additional epitope tags for detection and purification. The sequence encoding EGFR VHH was synthesized and inserted into pET32(a+) vector by Guangzhou IGE Biotechnology Ltd (China). The plasmid was subsequently transformed into competent Shuffle T7 *E coli*. Following selection, the transformed bacteria were cultured in a shaking incubator till they reached an OD_600_ value of 0.5 after which they were induced with 0.1 mM Isopropyl β-D-1-thiogalactopyranoside (IPTG) overnight at 25 °C, 250 RPM. On the following day, the cells were collected, washed using a high-speed centrifuge (6000 × g, 15 minutes, 4 °C) and resuspended in an appropriate volume of binding buffer - 500 mM NaCl, 25 mM Tris-HCl, 1 mM phenylmethylsulphonyl fluoride sulfonyl fluoride (PMSF) (Abcam, United Kingdom), 5% glycerol. This suspension was subsequently passed through a high-pressure homogenizer at 1000 psi for 4-6 cycles. The resulting flowthrough was collected and centrifuged at 10,000 × g for 30 minutes at 4 °C after which the supernatant was carefully collected and filtered using 0.45 μm syringe filters (Millipore, USA). The protein of interest was purified from the lysate using an NGC QUEST-10 fast protein liquid chromatography system (Bio-Rad, USA).

Briefly, the sample was loaded into a 5 mL Ni-charged cartridge (Bio-Rad) equilibrated with binding buffer using a sample inlet, followed by ~10 CVs of washing with the binding buffer. Non-specific interactions were further removed via a brief wash with 3% elution buffer (500 mM NaCl, 25 mM Tris-HCl, 1 mM imidazole, 1 mM PMSF and 5% glycerol). Finally, the protein of interest was eluted using a linear imidazole gradient from 40 to 500 mM. 2 mL fractions were collected using a BioFrac^TM^ fraction collector (Bio-Rad). Fractions containing the protein of interest as determined using the built-in UV detector were pooled together and concentrated using a 3 kDa cutoff centrifugal filter (Merck Millipore, USA) (4000 × g in a swinging-bucket rotor) and filtered through a 0.22 μm membrane. The proteins were further purified using a HiLoad 16/600 Superdex 200 pg size exclusion chromatography column (GE Healthcare, USA) in TBS (150 mM NaCl, 50 mM Tris-HCl, pH 7.4), at a flow rate of 0.5 mL/minute. Fractions containing the target protein were identified at the correct UV280 peak and pooled together before concentrating to a stock concentration of 1 mg/mL. Protein concentration was obtained using Nanodrop (extinction coefficient: 68550 M^-1^ cm^-1^, size: 31.06 kDa). Expression and purification of the protein of interest were verified throughout the process using sodium dodecyl sulphate-polyacrylamide gel electrophoresis and Coomassie blue staining.

Following purification, the nanobody was biotinylated using a Biotinylation Kit (Fast, Type B) - Lightning-Link® (Abcam) following the manufacturer's instructions. To account for the smaller size of the nanobody, it was diluted to 0.4 mg/mL and added to the equivalent of 1 mg of biotin mix to ensure efficient biotinylation per molecule without detrimentally affecting functionality. A control nanobody raised against mCherry was also purified and biotinylated as described above 10.

### Enzyme purification and expression

A plasmid encoding an inactive form of OaAEP1-Cys247Ala, provided by Prof. Bin Wu (Nanyang Technology University), was transformed into competent BL21 (DE3) cells. Following a selection using Kanamycin, protein expression was induced using 0.4 mM IPTG at 16 °C, 250 RPM for 18 hours. Lysis and purification were conducted as described above. Following affinity purification, the inactive enzyme was incubated at a concentration of 1 mg/mL with an activation buffer containing 200 mM acetate buffer, pH 3.7, 1 mM EDTA (ethylenediaminetetraacetic acid), 0.5 mM TCEP (tris(2-carboxyethyl)phosphine hydrochloride) (Abcam) for 10 days at 4 °C to facilitate the ligase's maturation via cleavage of the inhibitory cap domain. The resulting activated enzyme was dialyzed in storage buffer at pH 4.5 using PBS supplemented with 10% glycerol and stored at a concentration of 20 μM at -80 °C.

### RBCEV surface functionalization and drug loading

For OaAEP1-mediated peptide ligation, RBCEVs were incubated for 3 hours in a solution with 2 μM enzyme and 200-500 μM of peptide at 25 °C in PBS at pH 7. RBCEVs were washed three times using centrifugation at 21,000 × g for 20 minutes. For further functionalization, RBCEVs were incubated with streptavidin (Abcam) at a final concentration of 0.1 mg/mL for 2 hours at room temperature. A 10-fold excess of streptavidin was utilized to discourage crosslinking of biotin probes between EVs. The conjugated RBCEVs were subsequently washed as described previously to remove unbound streptavidin before incubating with a molar excess of biotinylated antibody/nanobody of choice. Biotinylated monoclonal antibodies - biotin anti-human CD326 (EpCAM) antibody (BioLegend, USA Cat# 324216,) and biotin mouse IgG2b, κ isotype ctrl antibody (BioLegend Cat# 400304) were incubated at a final concentration of 0.1 mg/mL while biotinylated nanobodies were incubated at 0.05 mg/mL for 2 hours at room temperature or overnight at 4 °C. The resulting RBCEVs were washed three times to remove unbound antibodies before being used for downstream experiments.

For RNA loading, RNA (antisense oligonucleotides/siRNA) from GenePharma, China was loaded into RBCEVs using REG1 (Carmine Therapeutics, Singapore) or Exo-Fect (System BioSciences, USA) following the manufacturers' instructions. Following loading, free RNA molecules and REG1/Exo-Fect were removed using three rounds of centrifugation at 21,000 × g for 20 minutes.

RNA loading efficiency was quantified by dissociating 50 μg of RNA loaded EVs and lysing them in 1% Triton X-100 buffer on ice. The resulting homogenate was supplemented with 6 × DNA loading dye (New England Biolabs, USA), loaded onto 12% Native PAGE gel along with a serial dilution of input RNA and electrophoresed in TBE buffer for 1 hour. Unloaded EVs were also run as a control to account for any endogenous RNA that may affect downstream quantification. The gel was post-stained with GelRed Nucleic acid stain (Biotium, USA) for 30 minutes in the dark and imaged using a Bio-Rad ChemiDoc gel documentation system. The serial dilution was used to plot a standard curve, which in turn was used to determine the quantity of RNA loaded into 50 μg of EVs. The total quantity of loaded RNA was divided by the number of EVs present in 50 μg of EVs (which was determined using a Zetaview system as described previously).

### Western blot analysis

RBCEVs were lysed with radioimmunoprecipitation assay (RIPA) buffer (Thermo Fisher Scientific) supplemented with protease inhibitor cocktail (Biotool, USA) for 5 minutes on ice. Cell lysate was incubated with RIPA buffer on ice for 30 minutes followed by a brief centrifugation step to remove any cell debris. Subsequently, 50 μg protein from cell lysates or 100 μg protein from RBCEVs were separated in 10% polyacrylamide gels together with a protein standard (Precision Plus Protein™ Kaleidoscope, Bio-Rad). The proteins were transferred to a Immobilon-P polyvinylidene difluoride membrane (Merck Millipore) after which they were blocked with 5% non-fat milk in Tris buffered saline containing 0.1% Tween-20 (TBST) for one hour at room temperature. The membranes were probed overnight at 4 °C with primary antibodies mouse anti-GAPDH (A01020, Abbkine, USA) or mouse anti-hemoglobin α (sc-514378, Santa Cruz Biotechnology, USA) at a 1:1000 dilution. The specific secondary horseradish peroxidase (HRP)-conjugated antibodies (Vector Laboratories, USA) were applied for 1 hour at a dilution of 1:3000 at room temperature. For biotinylated peptide detection, the blot was incubated directly with Pierce™ High Sensitivity Streptavidin-HRP (Thermo Fisher Scientific, dilution 1:5000) for 1 hour at 4 °C. The blot was imaged using a Bio-Rad ChemiDoc gel documentation system.

To estimate the stability of peptides conjugated to RBCEVs, RBCEVs were ligated with a biotinylated peptide of choice and incubated in EV-depleted human plasma at 37 °C with gentle agitation. Samples were collected at 12-hour intervals, washed, quantified and 100 μg was loaded per well for analysis by western blot. Following image acquisition, the intensity of bands was quantified using ImageJ software v1.8.0 (National Institute of Health, USA). Reversibility of ligation was also assessed in a similar way to that described above.

To evaluate RBCEV uptake, cells (600,000 cells/1.5 mL) were seeded in a 6 well-plate and treated with 100 μg of RBCEVs. After 24 hours, cells were collected and processed as described above.

### Flow cytometry

For flow cytometric analysis of surface proteins, cells were washed in FACS buffer to remove media before incubation with fluorescent-conjugated antibodies Alexa Fluor 488 (AF488)-α-hEGFR antibody (BioLegend Cat# 352908), PE-α-EGFR antibody (BioLegend CAT# 352904), AF488-α-FLAG antibody (BioLegend Cat# 637318), APC-α-GPA antibody (BioLegend Cat# 306608), APC-α-EpCAM antibody (BioLegend Cat# 324208) or APC-α-CXCR4 antibody (BioLegend Cat# 306509) for 30 minutes on ice in the dark. Cells with Fc receptors were pre-incubated with 5 μL of Human TruStain FcX (BioLegend) as required. Cells were subsequently washed three times in FACS buffer, before being subjected to flow cytometry analysis using either a CytoFLEX LX, CytoFLEX S or CytoFLEX system (Beckman Coulter, USA). FCS files were analyzed using FlowJo V10 (FlowJo, USA). Cells were first gated using an FSC-A versus SSC-A plot, to exclude debris and dead cells. Single cells were subsequently gated via an FSC-width versus FSC-height plot, excluding doublets and aggregated cells. The fluorescent-positive population of cells were subsequently gated by targeted fluorescent channels, such as FITC for AF488, FAM or CFSE, APC for AF647 and PE for tdTomato.

### Single EV flow cytometry

Flow cytometric analysis was performed as per the MISEV2018 guidelines outlined by the International Society of Extracellular Vesicles 11. For analysis of single EVs using flow cytometry, EVs were stained with streptavidin-Alexa Fluor® 488 (Abcam) or Alexa Fluor® 488 AffiniPure donkey anti-mouse IgG (Jackson ImmunoResearch, USA). For assessing RNA loading, FAM-conjugated ASOs were loaded onto EVs and the FAM signal acquired using the 488 nm laser. EVs were washed twice in PBS, diluted 10,000-fold and analyzed using a NanoFCM system (NanoFCM, United Kingdom). The laser power was fixed at 8 mW and the SS decay at 10%. The sampling pressure was fixed at 1.0 kPa prior to acquisition and events were recorded for a duration of 1 minute for each sample. In addition to EV + antibody controls, reagent controls containing staining antibodies diluted accordingly in filtered PBS were compared to PBS only, to eliminate any contribution by aggregated antibodies/proteins. EV samples were lysed with Triton X-100 and reanalyzed after each run to ensure that events within the EV gate were actually caused by the presence of EVs and not by non-EV particles/aggregates.

### ELISA

Competitive ELISA was used to determine the copy number of peptides per EV. Briefly, purified streptavidin was coated on Immunoplates (SPL Life Sciences, Korea) by incubating 100 μL per well of 5 μg/mL streptavidin overnight at 4 °C. The wells were subsequently blocked using blocking buffer (1% bovine serum albumin (BSA) in PBST (PBS with 0.05% Tween-20)) for one hour at RT. The plates were washed with wash buffer (PBST) and incubated for two hours with either a serial dilution of free peptide or 25 μg of peptide ligated EVs that were lysed using RIPA buffer to solubilize proteins. The excess supernatant was discarded and the plate was washed three times with PBST. The plate was then incubated for 1 hour with 100 μL of biotinylated HRP at a concentration of 10 μg/mL. The excess HRP was discarded and the plate washed four times before being incubated with 100 μL of TMB solution. The reaction was quenched using 100 μL stop solution (1M H_2_SO_4_) and the absorbance measured at 450 nm. The relative decrease in signal across the standard curve of biotinylated peptide incubated wells was used to quantify the copy number of peptides in the EV sample. Nanoparticle tracking analysis obtained via a Zetaview system was used to determine the number of EVs present in 25 μg of each sample. Finally, the total number of peptides present in each well was divided by the number of EVs to obtain the copy number of peptides per single EV.

Sandwich ELISA was utilized to quantify the copy number of monoclonal antibodies per RBCEV. Immunoplates (SPL Life Sciences, Korea) were pre-coated with streptavidin overnight. The wells were subsequently blocked using blocking buffer (1% bovine serum albumin (BSA) in PBST) for 1 hour. Polybiotinylated antibody coated RBCEVs (2.5 μg and 5 μg) were lysed in Triton X-100 non-denaturing lysis buffer (Thermo Fisher Scientific), quantified, and incubated on streptavidin coated plates. A serial dilution of polybiotinylated antibody was also included as a standard curve for quantification. The plate was incubated overnight at room temperature to allow binding of the biotinylated antibodies to the streptavidin on the immunoplate. The plate was subsequently washed three times using PBST (PBS with 0.05% Tween-20) to remove unbound proteins. Excess buffer was removed by inverting and tapping on absorbent paper. The wells were incubated for two hours with 100 μL HRP-conjugated goat anti-mouse antibody (Vector Bioscience) diluted at 1:3000 in blocking buffer. The plate was washed three times with PBST to remove unbound antibody and incubated for up to 15 minutes with 100 μL/well of TMB solution. When an adequate blue signal developed, the reaction was quenched using stop solution and the absorbance measured at 450 nm. The value from the well incubated with 2.5 μg EVs was compared to that incubated with 5 μg EVs to ensure that the reading was approximately twice, ensuring that the binding capacity of the plate was not saturated.

The serial dilutions of antibody in a linear range were used to plot a standard curve, which in turn was used to determine the quantity of antibodies present on 2.5 and 5 μg of EVs respectively. This value was divided by the number of EVs incubated with each well (which was determined using a Zetaview NTA system as described previously) to obtain the number of antibodies per EV. For the quantification of nanobodies per EV, a similar method was utilized by substituting streptavidin with a His tag antibody and using a FLAG tag antibody for detection. This was possible because each epitope tag was included in the nanobody construct at the N-terminal and the C-terminal respectively.

### Cryo-Electron microcopy (Cryo-EM)

Lacey carbon EM grids (EMS) were glow-discharged for 20 s in air in a Harrick Plasma system. Then 4 μL of the aqueous solution of the sample was applied on to the carbon side of the EM grid, which was then blotted for 2.0 s and flash-frozen in liquid ethane using a Vitrobot system (Thermo Fisher Scientific). The grids were stored in liquid nitrogen and loaded into a Tecnai Arctica electron microscope (Thermo Fisher Scientific) operated at 200 kV, equipped with a Falcon III direct electron detector. Data was collected manually in low dose mode to minimize radiation damage during image acquisition. Images were obtained at 20,000 × and 70,000 × magnification with the defocus value in the range of 2 to 4 μm.

### Generation and maintenance of cell lines

Human lung cancer NCI-H358 cells, acute lymphoblastic leukemia CEM cells and pancreatic adenocarcinoma AsPC-1 cells were obtained from the American Type Culture Collection (ATCC, USA). Acute myeloid leukemia MOLM13 cells were obtained from DSMZ Collection of Microorganisms and Cell Cultures (Braunschweig, Germany). Breast cancer MCF10CA1a (CA1a)-Fluc-mCherry cells and 4T1-tdTomato cells were the gifts from Prof. Judy Lieberman (Harvard Medical School) and Dr. Tam Wai Leong (Genome Institute of Singapore), respectively. 4T1-tdTomato cells were transduced with a lentiviral vector encoding human EGFR. MOLM13 cells were transduced with pLV-Fluc-mCherry-Puro plasmid. Successfully transduced cells were selected for using puromycin to establish stable cell lines. All cell lines were maintained in DMEM or RPMI (Thermo Fisher Scientific), supplemented with 10% heat inactivated fetal bovine serum (FBS) and 1% penicillin/streptomycin (Thermo Fisher Scientific, USA). The cells were maintained at 37 °C in a humidified atmosphere containing 5% of CO_2_.

### Human T cell isolation and activation

Human peripheral blood mononuclear cells (PBMCs) were obtained via isolation from apheresis samples obtained from the Health Sciences Authority of Singapore from individuals with informed consent. Briefly, the blood was diluted 2-fold in PBS and layered gently over 4 mL of Ficoll-Paque plus (Cytiva, USA). The cells were centrifuged at 400 × g at 18 °C for 30 minutes with the brake turned off. The PBMCs at the interface above the Ficoll-Paque layer were carefully transferred into a sterile tube, diluted in PBS and washed twice at 300 × g at 18 °C for 8 minutes. The resulting pellet was resuspended in MACS buffer (Miltenyi Biotec, Germany) at a concentration of 10^8^ cells in 800 µL. Monocyte depletion was performed by incubating the cell suspension with 200 µL of CD14 microbeads (Miltenyi Biotec) and performing negative selection on a MS column (Miltenyi Biotec). The flow through, depleted of CD14-positive monocytes, was subsequently spun down. The resulting pellet was resuspended in 160 µL of MACS buffer and incubated with 40 µL of CD4 microbeads (Miltenyi Biotec) for 15 minutes on ice. The cells were then pipetted onto a pre-equilibrated MS column on a magnetic separator (Miltenyi Biotec). Unlabeled cells were discarded and the column was rinsed before the CD4-positive T cells were eluted by removing the column from the rack and using the plunger to force out the magnetically labelled cells. The cells were recovered overnight in TexMACS media (Miltenyi Biotec).

For T cell activation, the CD4-positive T cells were incubated with CD3/CD28 Dynabeads (ThermoFisher) at a 1:1 ratio of beads:cells. The cells were maintained in TexMACS supplemented with 30U/mL rIL-2 (Miltenyi Biotec) and allowed to expand under optimal culture conditions. Activator Dynabeads were removed by vigorous pipetting 24 hours after activation.

### Binding and uptake assays for RBCEVs

For the cell association assay, 100,000 cells were incubated with 10-20 μg RBCEVs in 500 μL growth medium on an end-over-end shaker for 1 hour at 4 °C. The cells were washed twice with PBS and incubated with 1 μL APC-anti-GPA antibody (BioLegend Cat# 306608) for 1 hour at 4 °C, washed three times with PBS and analyzed by flow cytometry.

To assess *in vitro* targeting activity, cells were seeded in 24-well plates (200,000 cells/0.5 mL) and kept in optimal growing conditions for 24 hours. Afterwards, the cells received 2 μg of CFSE-labelled EV (for EGFR/EpCAM targeted) or 50 μg of CFSE-labelled EVs (for CXCR4 targeting) for 2 hours at 37 °C, then washed twice with cold PBS/FACS buffer and analyzed immediately by flow cytometry.

To further validate the specificity of targeting, the cells were preincubated with 200 μM of free T140 peptide for 1 hour. After washing once with PBS, the cells received 50 μg of T140-ligated CFSE-labelled RBCEVs for 1 hour at 37 °C, then washed twice with cold PBS and analyzed immediately by flow cytometry.

### Cell proliferation and apoptosis assays

The effect of T140-KLA EVs on cell proliferation was assessed by CCK8 assay (Biosharp, China). Briefly, MOLM13 cells seeded in 24-well plates (50,000 cells/0.5 mL) received 50 μg of RBCEVs and were maintained under optimal growing conditions. After 24, 48, 72 and 96 hours of treatment with RBCEVs, 10% (vol/vol) of CCK8 reagent was added to the plate for 2 hours at 37 °C, protected from light. The absorbance of collected supernatants was read at 450 nm using a SynergyTM H1 microplate reader (BioTek, USA) or a Spark 10 M microplate reader (Tecan, Switzerland). To assess the viability of breast cancer cells, 10,000 CA1a or 4T1-tdTomato-hEGFR cells were seeded in 96-well plates 24 hours prior to addition of miR-125b-ASO-loaded RBCEVs. Following 3 days of incubation with EVs, cell viability was assessed as described above.

Annexin V-FITC and propidium iodide (PI) staining (Nanjing Vazyme Biotech, China) were used to determine apoptosis of treated cells. Briefly, after 96 hours of treatment with RBCEVs, MOLM13 cells were collected and washed twice with cold Annexin V binding buffer, then incubated with 100 μL of binding buffer containing 5 μL of annexin-V-FITC and 5 μL of PI for 10 minutes at room temperature, protected from light. The cells were subsequently washed twice with cold binding buffer and analyzed by flow cytometry.

### Reactive oxygen species and mitochondrial membrane potential assessment

MitoTracker Red CM-H2XRos (Thermo Fisher Scientific) and CM-H2DCFDA (Thermo Fisher Scientific) were used to assess the mitochondrial membrane potential (MMP) and quantify reactive oxygen species (ROS) respectively. Briefly, MOLM13 cells, seeded in 24-well plates (200,000 cells/0.5 mL), received 50 μg of RBCEVs with or without peptide coating and maintained in optimal culture conditions for 24 hours. Following the treatment, the cells were collected, washed with PBS and probed with MitoTracker Red (final concentration 50 nM) or CM-H2DCFDA (final concentration 1 μM) and incubated at room temperature for 30 minutes, protected from light. The cells were subsequently washed twice with cold PBS and analyzed using flow cytometry as described above. The fluorescent-positive population of cells was gated using the appropriate channels (PE for MitoTracker Red CM-H2XRos and FITC for CM-H2DCFDA).

### Immunofluorescent imaging to track EV/ASO uptake

Cells were pre-seeded on poly-D-lysine (Gibco, USA) coated 12 mm coverslips (Citoglasss, China) 24 hours prior to treatment. Following treatment with RBCEVs, the coverslips were rinsed with fresh media, and stained with CellMask membrane dye (Thermo Fisher Scientific) for 10 minutes at 37 °C. Cells were rinsed twice in PBS and stained with Hoechst 33342 (Abcam) for 5 minutes at room temperature. The coverslips were rinsed three times with PBS before being fixed for 12 minutes using 4% paraformaldehyde in PBS (Alfa Aesar, USA). The coverslips were subsequently washed three times with PBS followed by a final wash with MilliQ water before being mounted on slides using anti-fade fluorescence mounting medium (Vector Biolabs, USA). Images were acquired using an inverted Zeiss LSM710 confocal microscope or an Olympus FV3000 confocal microscope. Image acquisition was conducted using Zeiss Zen software (2011) or FluoView software while further analysis and quantification was conducted using ImageJ software. Cell areas were selected as regions of interest (ROIs) based on the CellMask signal. CFSE/FAM signals were measured as mean pixel intensity in these selected ROIs. Total measurement area covered ~1000 cells per condition.

### RNA extraction and RT-qPCR

RNA was extracted from cells or tissues using TRIzol (Thermo Fisher Scientific) according to the manufacturer's instructions. Following isolation, the RNA was resuspended in RNase-free water and the RNA concentration was quantified using a NanoDrop analyzer (Thermo Fisher Scientific). Extracted RNA was reverse transcribed using a cDNA reverse transcription kit (Thermo Fisher Scientific) following the manufacturer's protocol. Subsequently, miR-125b levels were quantified using a TaqMan® miRNA assays kit (Thermo Fisher Scientific) and the expression level normalized to a suitable internal control (U6B for cells of human origin, and snoRNA234 for mouse cells). RT-qPCR was carried out using a QuantStudio 6 Flex (Applied Biosystems, USA).

For the quantification of miR-125b ASO per EV, a Taqman real-time PCR assay (ThermoFisher ID #007655_mat) was used. In brief, RNA extracted from 100 μg of miR-125b ASO-loaded RBCEVs was compared to a serial dilution of miR-125b ASOs using cycle threshold (Ct) values to obtain the number of ASO molecules present in the EVs. RNA extraction and RT-qPCR were conducted as described above. Concurrently, nanoparticle tracking analysis was used to determine the number of EVs present in the 100 μg of EVs used for RNA extraction. These data were used to obtain the number of ASOs per EV.

### Hemolysis assay

RBCEVs (0.5 mg/mL) uncoated or conjugated with a biotinylated control peptide (B-Peptide) or VHH were incubated with fresh human whole blood at 37 °C for 1 hour. Intact RBCs were then pelleted by centrifugation at 800 × g for 5 minutes followed by centrifugation at 21,000 × g for 20 minutes to remove RBCEVs. The resulting supernatant was assessed for Hemoglobin content using a hemoglobin quantification assay kit (Abcam). Incubation with 0.01-1% Triton X-100 is included for reference and degree of hemolysis for each condition is represented as a percentage compared to the positive control (incubation with ACK lysis buffer).

### Generation of *in vivo* cancer xenograft models

All *in vivo* experiments were conducted according to protocols approved by the Institutional Animal Care and Use Committee under the National University of Singapore and the Animal Ethics Committee at the City University of Hong Kong. NOD-SCID Gamma (NSG) mice with or without SGM3 cytokine combination were obtained from the Jackson Lab (USA) and bred in our facilities. Mice of similar ages were tagged and grouped randomly for control and test treatments. Experiments were performed in a blinded manner.

To generate an acute myeloid leukemia (AML) xenograft mouse model, 8-week old NSG mice were preconditioned intraperitoneally (i.p.) with 20 mg/kg of Busulfan (Sigma-Aldrich). MOLM13-luciferase cells (300,000 cells in 0.25 mL of PBS) were injected into the tail vein of the mice. After 7-10 days, the bioluminescence signal was detected using a Lumina II *in vivo* imaging system (IVIS) (PerkinElmer, USA) following an i.p. injection of 150 mg/kg of D-luciferin (PerkinElmer). Mice with similar bioluminescence signals were selected for subsequent experiments.

Lung metastatic breast cancer allografted mouse models were generated by intravenously injecting NSG-SGM3 mice with 500,000 4T1-tdTomato-hEGFR cells in PBS. Tumor cells engrafted in the lung were detected using flow cytometry by gating the tdTomato-hEGFR double-positive population of cells.

### Administration of RBCEVs

To assess the uptake of RBCEVs by circulating leukemia cells, mice with established MOLM13 leukemia xenografts received a single dose of intravenously injected CFSE-labelled RBCEVs (40 mg EVs/kg) with T140 or TL5 peptide coating. After 4 hours, blood samples were collected using retro-orbital bleeding (non-lethal) into heparinized tubes. After centrifugation (1500 × g for 5 minutes at 4 °C), the supernatant (plasma) was discarded, the cells were passed through a 70 μm cell strainer and incubated in ACK lysis buffer on ice for 20 minutes. The resulting pellet was washed twice (1500 × g for 5 minutes at 4 °C) in cold PBS and resuspended in FACS buffer for flow cytometric analysis. The cells were gated based on FSC-A and SSC-A to exclude debris and dead cells, FSC-width vs. FSC-height gates to exclude doublets and aggregates and the PB450 channel was used to exclude SYTOX blue dead cell stain (Invitrogen, USA) positive cells. Subsequently, mCherry-positive leukemia cells were gated using the ECD channel and the FITC channel was used to obtain the percentage of CFSE-positive cells within the mCherry-positive cell population.

For the treatment of AML xenografted mice, the mice received 40 mg/kg intravenous injection of RBCEVs with KLA, T140 or T140-KLA peptide conjugation every 2 days. The bioluminescent signal was also measured every two days using IVIS as described above.

For intratracheal delivery of RBCEVs, mice were initially anesthetized with isoflurane. Anaesthetized mice were immobilized on a stand at an 80° angle, their tongues were pulled out using sterile forceps while their noses were covered to prevent breathing through the nose. The EVs suspension (400 µg in a volume of 50 µL) was then pipetted into the buccal cavity above the tracheal opening and the mice were allowed to breathe in the administered EVs. Once the mice had taken 20 successful breaths, they were laid on their back for a duration of 15 minutes on anesthesia to prevent any residual EVs from being breathed out.

Targeted delivery of fluorescent RNAs to tumor bearing mice and analysis of relative miR-125b knockdown in tumor cells was performed in tumor-bearing mice 5 days after inoculating with the cancer cells when their tumor burden was significantly high. Mice were sacrificed at the 8-hour timepoint after EV administration for assessing relative EV uptake of targeted and non-targeted EVs. For the assessment of miR-125b knockdown, the mice were sacrificed 36 hours after EV administration and the tissues processed as described above for RT-qPCR analysis. Treatment using ASO-loaded EVs was started 24 hours post-inoculation. Mice were treated daily for 6 days and sacrificed on day 7, their lungs excised and homogenized using a gentleMACS™ dissociator (Miltenyi Biotec, Germany). Homogenates were passed through a 70 μm cell strainer, incubated with ACK RBC lysis buffer for 20 minutes and analyzed by flow cytometry for tdTomato fluorescence as described above.

### Fluorescent activated cell sorting (FACS)

FACS was utilized to sort tumor cells from mouse lung cells to assess relative knockdown in each cell type 36 hours following administration of different ASO-loaded EV treatments. Lung homogenates were treated as described above to obtain a single cell suspension. Cells were resuspended in FACS buffer containing 10% FBS and 2 mM EDTA to prevent clumping of cells. Cell were subsequently stained with an APC-conjugated anti-hEGFR antibody (BioLegend) and run on a MoFlo® Astrios™ sorter (Beckman Coulter), sorting 250,000 tumor and non-tumor cells into separate tubes for RT-qPCR analysis. Tumor cells were distinguished by tdTomato and APC double-positive fluorescence.

### Histopathology

For H&E staining, the spleen was collected from leukemic mice following 14 days of RBCEV treatment. Tissues were stored in 10% neutral buffered formalin solution (Sigma-Aldrich) overnight and transferred to 70% ethanol. Tissues were dehydrated in increasing concentrations of ethanol (70%, 95%, 100%) and washed in three baths of Histo-Clear solution (National Diagnostics) at RT. The tissues were subsequently impregnated in 2 baths of paraffin wax (Leica Biosystems) each for 2 hours at 58 °C. Subsequently, the tissues were embedded in paraffin and sectioned at a thickness of 5 µm using a microtome (Leica RM2255). Sections were dried in an incubator at 37 °C.

For dewaxing, dried slides were incubated in 3 baths of Histo-Clear followed by a 10-minute incubation in 50% Histo-Clear and 50% ethanol. Slides were subsequently immersed in absolute ethanol, and solutions of 90%, 75% and 50% ethanol before being rehydrated in water. Rehydrated sections were stained with an H&E Staining Kit (Abcam) according to the manufacturer's instructions. Slides were dehydrated in three baths of absolute ethanol and cleared in a bath of Histo-Clear for 10 minutes before being mounted using Histomount (National Diagnostics, USA). Sections were imaged using a TissueFAXS microscope (TissueGnostics, Austria) at 400 × magnification. Percentage of leukemic infiltration in spleen sections was quantified using ImageJ by determining the percentage of leukemic cell nuclei based on their distinct morphology.

### Immunohistochemistry

To assess the EV uptake and area of tumor metastases in the lungs of mice, lungs were excised with the trachea intact, inflated with OCT using a blunt-end needle and flash frozen in OCT. Frozen tissue blocks were sectioned at a thickness of 8 µm, fixed with 4% PFA (15 min), blocked with 10% normal donkey serum and stained with anti-CD31 antibody (ab28364) (Abcam) and anti-alpha smooth muscle actin antibody (ab7817) (Abcam). Sections were counterstained with Hoechst 33342 and mounted and imaged using an Olympus FV3000 confocal microscope with FluoView software. ImageJ was used to quantify the percentage tumor area via the detection of endogenous tdTomato fluorescence across three intact parasagittal sections of the lung. Similarly, the mean CFSE signal in tumor nodules was used to quantify EV uptake by tumor cells in each condition from ten randomly chosen tile scans of the lung. Images for quantification were captured in a blinded manner using the Hoechst channel.

To assess the delivery of EVs to different regions of the lung, following administration of a single dose of CFSE-labelled EVs, the lungs were frozen vertically in OCT with the trachea positioned on top as described above. The lungs were then sectioned at 10 µm thickness starting from the top, with sections being taken approximately every 500 µm until the bottom of the lung was reached. The sections were then analyzed using ImageJ to detect the percentage coverage of lung area by the CFSE-positive EVs.

### Toxicity and immunogenicity assessment

Uncoated RBCEVs or RBCEVs coated with a control peptide or VHH, were injected into the tail vein of 7-week old female C57BL/6 mice at a dosage of 40 mg EVs per kg. The mice were regularly monitored during the study for visible signs of toxicity or stress. Blood samples were collected at 0 and 24 hours after the injection. IL-6 and TNF concentrations in the blood were measured using IL-6 and TNF ELISA kits (Elabscience, China) according to the manufacturer's instructions. Blood samples collected at the end point (24 hours) were analyzed for biochemistry parameters including the concentration of alanine aminotransferase (ALT), aspartate aminotransferase (AST), alkaline phosphatase (ALP), creatinine, total Bilirubin and creatine kinase (CK) on a Cobas 6000 (Roche Diagnostics, Switzerland). RNA samples were isolated from the liver for RT-qPCR analysis of immune related genes including *IL-6, Ifna4, Ifng*, and *Osal2.*

The primer sequences used for RT-qPCR are as follows:

- *IL-6* 5'-GCCAGAGTCCTTCAGAGAGATA-3';
- 5'TCTGTGACTCCAGCTTATCTGTTA-3';
- *Ifng* 5'-GATTGCGGGGTTGTATCTGG -3';
- 5'-ACTGCAGCTCTGAATGTTTCTT-3';
- *Tnf* 5'- ACCGTCAGCCGATTTGCTAT -3';
- 5'- CCGGACTCCGCAAAGTCTAA-3';
- *Eif2ak2* 5'- GGAGTCCGCCGGGAAAA -3';
- 5'- TTTTCCTCCCAGTGGCCAAA-3';
- *Ifnb1* 5'- TTCCTGCTGTGCTTCTCCAC -3';
- 5'- GGAGCTCCTGACATTTCCGA-3';
- *Ifna4* 5'-CTGGTAATGATGAGCTACTACTGG-3';
- 5'-CCTTCTCCAAGGGGAATCCAA-3';
- *Ifna11* 5'-GGTCCTGGCACAAATGAGGA-3';
- 5'- TCCAAGCAGCAGATGAGTCC-3';
- *Ifna12* 5'-AAGACTGAGTGAGAAGGAGTGAG -3';
- 5'- GAGATGCCAGAATTTGAGCAGTG-3';
- *Osal2* 5'-TTCCATGCAACTCTCCATCCCAT-3';
- 5'-TTGTCCCCTTTCCCGAGGAG -3';
- *Rsad2* 5'-ACACCCTCCAATTACTGCTGAC-3';
- 5'- GGCTGGGACCATGAACAAACAG -3';
- *Gapdh* 5'-AGGTCGGTGTGAACGGATTTG-3';
- 5'-TGTAGACCATGTAGTTGAGGTCA-3'.

For the assessment of potential hepatotoxicity induced by T140-KLA-EVs, unmodified EVs or T140-KLA peptide-conjugated EVs were injected into NSG mice with established MOLM13 xenografts. Blood samples were analyzed 24 hours post-injection for AST, ALT, ALP and bilirubin levels as described above.

### Statistical analysis

GraphPad Prism 8 was used to conduct all statistical analyses. One-tailed Student's t-test was used to assess significance levels between controls and experimental samples. For analyzing the difference among multiple treatment groups, two-way ANOVA was utilized. Throughout this study, a p-value <0.05 was considered to be significant. Data in the graphs are represented as the mean, with error bars indicating the standard error of the mean (SEM). Each experiment was repeated at least three times using different batches of RBCEVs and/or cells from different passages. Animal experiments were performed in groups of 3 to 4 mice.

## Results

### Enzyme-mediated peptide ligation facilitates the stable introduction of functional molecules onto the RBCEV surface

**Figure 1:**
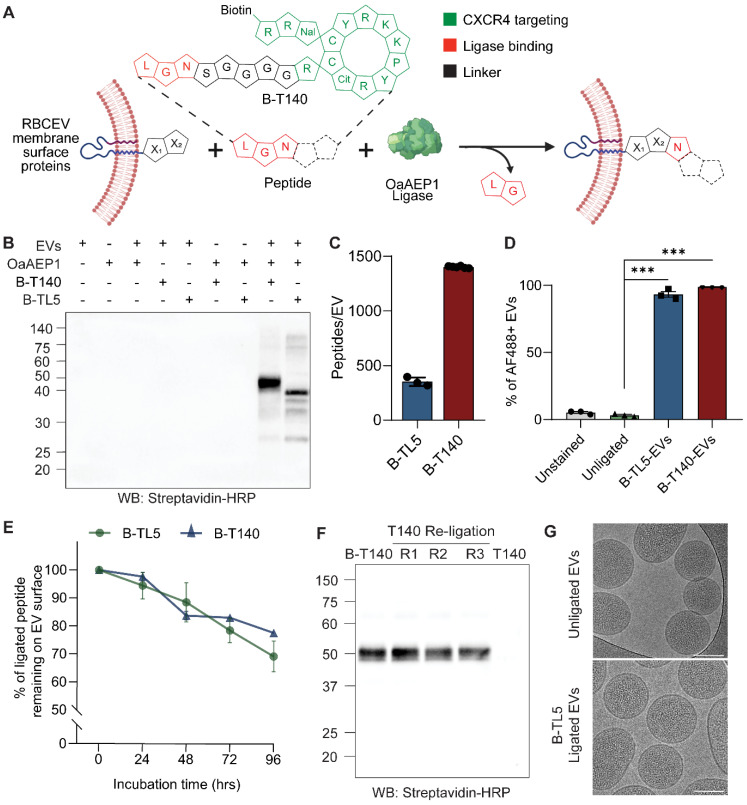
** OaAEP1 ligase stably conjugates peptides onto the RBCEV surface. (A)** Outline of the ligation reaction catalyzed by OaAEP1-Cys247Ala, where peptides with functional groups on the N-terminal are covalently ligated at the C-terminal to EV proteins. The inset provides a schematic representation of B-T140 peptide. The single letter code specifies amino acids responsible for targeting activity (green) or ligation (red). **(B)** Western blot (WB) analysis of B-T140 and B-TL5 peptides ligated to RBCEVs. **(C)** Copy number of B-TL5 and B-T140 peptides per EV determined using competitive ELISA (n = 3-6 biological replicates). **(D)** Percentage of peptide ligated EVs obtained via single-EV flow cytometric analysis of B-TL5 and B-T140 peptide conjugated RBCEVs, probed for biotin using streptavidin-AF488(n = 3 replicates). **(E)** Summary of western blot analysis assessing the stability of OaAEP1-mediated conjugation, based on the percentage of remaining peptide on RBCEVs that were ligated with B-TL5/B-T140 and incubated in EV-depleted human plasma at 37°C for 24-96 hrs (n = 3 replicates for each timepoint).** (F)** Irreversibility of OaAEP1 mediated ligation: B-T140 ligated RBCEVs were re-ligated with unbiotinylated T140 peptide repeatedly (repeat R1-3) and analyzed by western blot. The last lane illustrates EVs directly ligated with unbiotinylated T140 for reference. **(G)** Representative cryo-EM images of unligated and peptide-ligated RBCEVs. Scale bar, 100 nm. In **B** and **F**, the abundance of biotinylated peptide-conjugated proteins were quantified by the level of biotin detected using streptavidin-HRP. Molecular weights (kDa) of the protein marker are shown on the left of each blot. For quantification of peptides per EV in **C**, NTA was used to obtain the number of input EVs, thereby providing an estimate of peptide copy number per EV. The graphs present the mean ± SEM. Student's one-tailed t-test ***P < 0.001.

We employed OaAEP1 Cys247Ala, a previously reported asparaginyl peptidase that demonstrates efficient ligation of proteins and peptides, to covalently ligate peptides onto existing RBCEV membrane proteins 12. These peptides were synthesized with a NGL motif at the C-terminal to facilitate intermediate formation with the enzyme, while a linker sequence (GGGGS) separated the ligation motif from the functional domain. Following incubation of the enzyme-peptide intermediate with RBCEVs, the C-terminal -GL residues were cleaved by the enzyme and the remainder of the peptide transferred onto a RBCEV membrane protein displaying a suitable N-terminal recognition motif, resulting in the formation of a covalent peptide bond (Figure 1A). As per previous reports, the N-terminal amino acids X_1_ and X_2_ of the substrate are most preferably L and R/G respectively, though alternate variations have also been shown to be functional, albeit displaying variable efficiency 13.

The functional domain of the peptide was included at the N-terminal and involved the addition of simple functional groups such as biotin (B) for detection/further functionalization or extended as peptide sequences for targeting or therapeutic effect (Table S1). We have previously demonstrated that direct ligation of simple peptides such as B-TL5 or an EGFR-targeting peptide yielded on average ~380 copies of peptides per RBCEV 8. Interestingly, in this study we discovered that certain peptides such as a CXCR4-targeting peptide (T140) and its scrambled form (Scr-T140) that possessed a stable cyclic conformation (Figure 1A inset, Figure S1A) were ligated more efficiently onto the RBCEV surface. Western blotting revealed that these cyclic peptides yielded significantly higher copy numbers of peptides per RBCEV as shown by the thicker bands of ligated RBCEV proteins at ~50 kDa (Figure 1B, Figure S1B). The copy number of peptides ligated per RBCEV for the B-T140 peptide was dependent on the pH, temperature and peptide concentration in the ligation reaction, as reported previously (Figure S1C) 8. Competition ELISA of B-T140-EV lysates revealed that each EV was conjugated with approximately 1402 peptides, an approximately 3-fold increase over the reported copy number for B-TL5 peptide (Figure 1C). Of note, competition ELISA of B-TL5-EVs yielded a copy number of ~351 peptides per EV, a value in close proximity to the previously reported copy number of 380 obtained via western blotting (Figure 1C) 8. Further verification of copy number was conducted using western blot via the comparison of biotin signals from B-T140 peptide coated RBCEVs with the signal from a serial dilution of dibiotinylated HRP revealed the presence of over 1000 copies of the cyclic peptide per RBCEV, an observation that was in accordance with our ELISA data (Figure S1D-E). Moreover, we found the same spectrum of ligated protein bands when B-T140 peptide was ligated onto RBCEVs purified from three independent donors, suggesting that RBCEVs from each donor expressed the same surface proteins that were consistently ligated with the B-T140 peptide, confirming the reproducibility of our approach (Figure S1D-E).

We further confirmed the increased efficiency of the B-T140 peptide ligation using single-EV flow cytometry. RBCEVs were ligated with either B-TL5 or the B-T140 peptide and the biotin tag was detected on the EV surface using Alexa Fluor 488-conjugated streptavidin via a NanoFCM system. Single EVs were gated out as a distinct population from the background as shown in figure S2A. The single EV flow cytometry data showed that the entire EV population increased in AF488 fluorescence upon ligation with either peptide, indicating that on average ~95% and ~99% of EVs were positive for biotin from the B-TL5 and B-T140 conjugation respectively (Figure 1D). However, a comparison of the mean fluorescent intensity revealed that the B-T140 ligated EVs exhibited a greater than 2-fold increase in fluorescent intensity compared to B-TL5 ligated EVs (Figure S2B). The single EV flow cytometry plots with all necessary controls (detergent control, reagent control) along with detailed experimental settings are also provided as per MISEV2018 and MIFlowCyt-EV guidelines (Figure S2C) 11, 14. These data demonstrate that the OaAEP1-ligase-mediated conjugation was significantly more efficient with the newly designed T140 peptides.

We also sought to test the stability of this enzyme-mediated conjugation method to ensure that surface modified EVs remain functional *in vivo* following systemic administration. To mimic *in vivo* conditions, we ligated RBCEVs with either B-TL5 or B-T140 and incubated them with human plasma at 37 °C. Samples were analyzed at 24-hour intervals by western blot. Analysis revealed that more than 70% of each peptide remained intact on the EV surface after 96 hours, confirming the stable nature of the conjugation (Figure 1E, S3A-B). The partial degradation of peptides in plasma can be attributed to the presence of proteases that may exacerbate the degradation process of both the peptides and the EV membrane proteins they are ligated to. Despite the slight degradation observed, the data indicates that this conjugation approach is sufficiently stable for EVs to retain ligated peptides in a functional state for successful *in vivo* applications. Moreover, the enzymatic ligation was demonstrated to be largely irreversible, as evidenced by the undiminished bands of biotinylated peptide ligated EVs, even after repeated ligation with a non-biotinylated form of the same peptide (Figure 1F, S3C).

Cryogenic electron microscopy (cryo-EM) images revealed that ligated and unligated RBCEVs exhibited similar and intact morphology, confirming the non-invasive nature of the ligation process that conserved the EVs' structure and integrity (Figure 1G). In addition, we investigated if enzyme-mediated surface modification changed the physicochemical properties of the EVs. Characterization of unligated or ligated RBCEVs using nanoparticle tracking analysis and dynamic light scattering analysis showed that there was no significant difference in the hydrodynamic diameter, zeta potential, polydispersity index and size distribution profiles before and after the enzymatic reaction (Figure S3D-G). These data suggest that surface modification using our enzymatic method does not affect the physicochemical properties of the RBCEVs including size, surface charge and colloidal stability.

### Ligation of CXCR4-targeting T140 peptide enhances EV accumulation in CXCR4-positive cells

CXCR4 is a G protein-coupled receptor whose overexpression is associated with the onset or exacerbation of many human cancers 15. As determined using flow cytometry analysis, acute monocytic leukemia MOLM13 cells and acute lymphoblastic leukemia CEM cells overexpressed CXCR4 on their surface while human pancreatic adenocarcinoma cells were negative for human CXCR4 (Figure S4A). For functional peptide-mediated CXCR4 targeting, we adopted from the literature the cyclic T140 peptide, a well-known CXCR4 binding peptide 16. The T140 peptide was subsequently modified as illustrated in figure 1A to facilitate the ligation reaction (sequence shown in Table S1). As described above, RBCEVs were ligated with T140 peptide at high efficiency, resulting in well over 1000 copies of peptide per EV.

**Figure 2:**
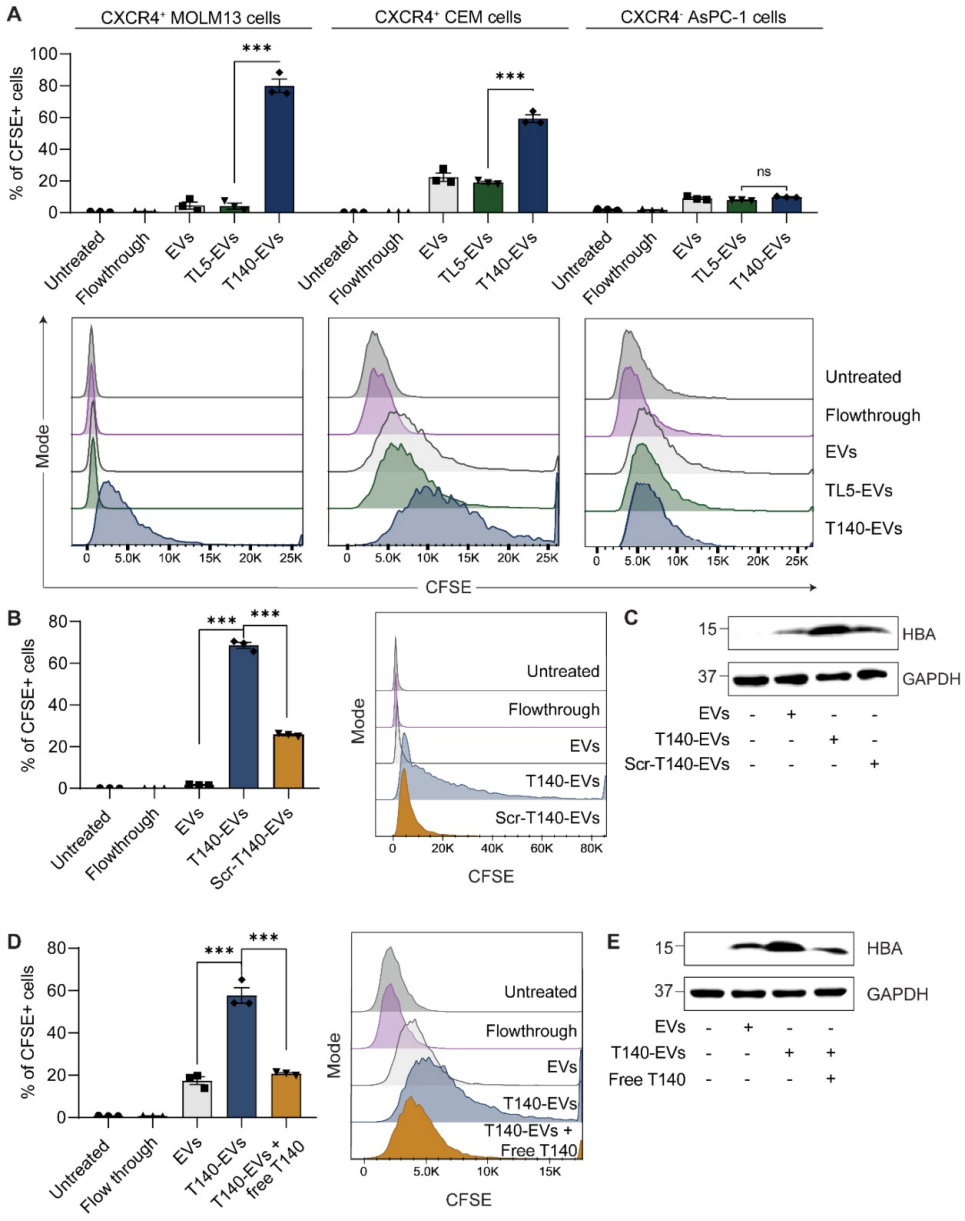
** Ligation of RBCEVs with a T140 peptide allows CXCR4-specific cellular targeting. (A)** *In vitro* uptake of control RBCEVs or RBCEVs ligated with CXCR4-binding T140 peptide in three cell lines with varying CXCR4 expression, presented as the percentage of cells that uptake CFSE-labelled RBCEVs, quantified using flow cytometry. **(B)** Comparison of uptake of T140 or Scr-T140 peptide ligated RBCEVs by MOLM13 cells based on flow cytometry analysis of CFSE. **(C)** Western blot of hemoglobin A (HBA) to track EV uptake in MOLM13 cells incubated with targeting T140-EVs or non-targeting Scr-T140-EVs. **(D)** Uptake of T140-ligated RBCEVs by MOLM13 cells pretreated with free T140 peptide. **(E)** Western blot analysis of HBA indicating RBCEV uptake in MOLM13 cells in the presence or absence of a pretreatment with free T140 peptide. For **A**, **B** and **D**, graphs include data from 3 biological replicates prepared from RBCEVs from independent donors. The graphs present the mean ± SEM. Student's one-tailed t-test: ns - not significant, *** P < 0.001. For **C** and **E**, EV uptake was tracked via HBA while GAPDH served as an internal control.

To test the ability of the T140 peptide to induce RBCEV uptake specifically in CXCR4-positive cells, we labelled unligated and peptide-ligated RBCEVs with CFSE, a membrane-permeant fluorogenic dye. Upon entering EVs, the diacetate group of CFSE is cleaved by intraluminal esterases, giving rise to the fluorescent ester which shows greatly diminished membrane permeance. The cleaved CFSE molecules are subsequently fixed to intraluminal proteins via stable amide bonds through its succinimidyl group, allowing accurate EV tracking. Leukemic MOLM13 and CEM cells were incubated with CFSE-labelled RBCEVs for 2 hours. The *in vitro* cellular uptake was analyzed by flow cytometry analysis of CFSE signals. As shown in Figure 2A, the ligation of the T140 peptide significantly improved the internalization of CFSE-labelled EVs into MOLM13 and CEM cells, but not CXCR4-negative AsPC-1 cells, indicating effective CXCR4 specific targeting. Moreover, the CFSE signal from cells treated with control peptide ligated EVs (TL5-EVs) was comparable to that of unmodified EVs (Figure 2A), indicating that the increase in uptake of T140-EVs can be attributed to the presence of the T140 peptide and was not due to the effect of ligation. To demonstrate that the presence of CFSE fluorescence in cells was due to the uptake of EVs and not due to the transfer of free dye, we also included a flowthrough control, where an equivalent volume of supernatant from the last EV wash resulted in no significant increase in cellular fluorescence.

Furthermore, ligation of a scrambled T140 peptide (Scr-T140) onto EVs resulted in significantly lower EV uptake in MOLM13 cells as compared to the ligation of the CXCR4-targeting T140 peptide (Figure 2B). Of note, Scr-T140-EVs showed higher internalization than that of unmodified EVs. In this regard, we presumed that either the scrambled sequence still partially interacted with CXCR4 or that the amino acid positioning, in particular the arginine backbone (R5, R6, R7) (Figure S1A), improved the cellular penetrating ability of the sequence, resulting in enhanced EV internalization. In addition, to further confirm that the uptake of EVs occurred via the specific interaction of EV-ligated-T140 and CXCR4, we demonstrated that a pre-treatment of MOLM13 cells with free T140 peptide abrogated the T140-mediated increase in EV uptake (Figure 2D). Western blot analysis of MOLM13 cell lysates following RBCEV treatment also showed a clear increase of hemoglobin A (HBA), a specific marker of RBCEVs, when treated with T140-EVs compared to Scr-T140-EVs or antagonist pre-treated cells (Figure 2C, E). Taken together, this data suggests that T140 peptide ligation is capable of increasing EV uptake specifically in CXCR4-positive cells.

### Conjugation of EVs with a bifunctional peptide with a pro-apoptotic domain confers CXCR4-specific cytotoxicity

**Figure 3:**
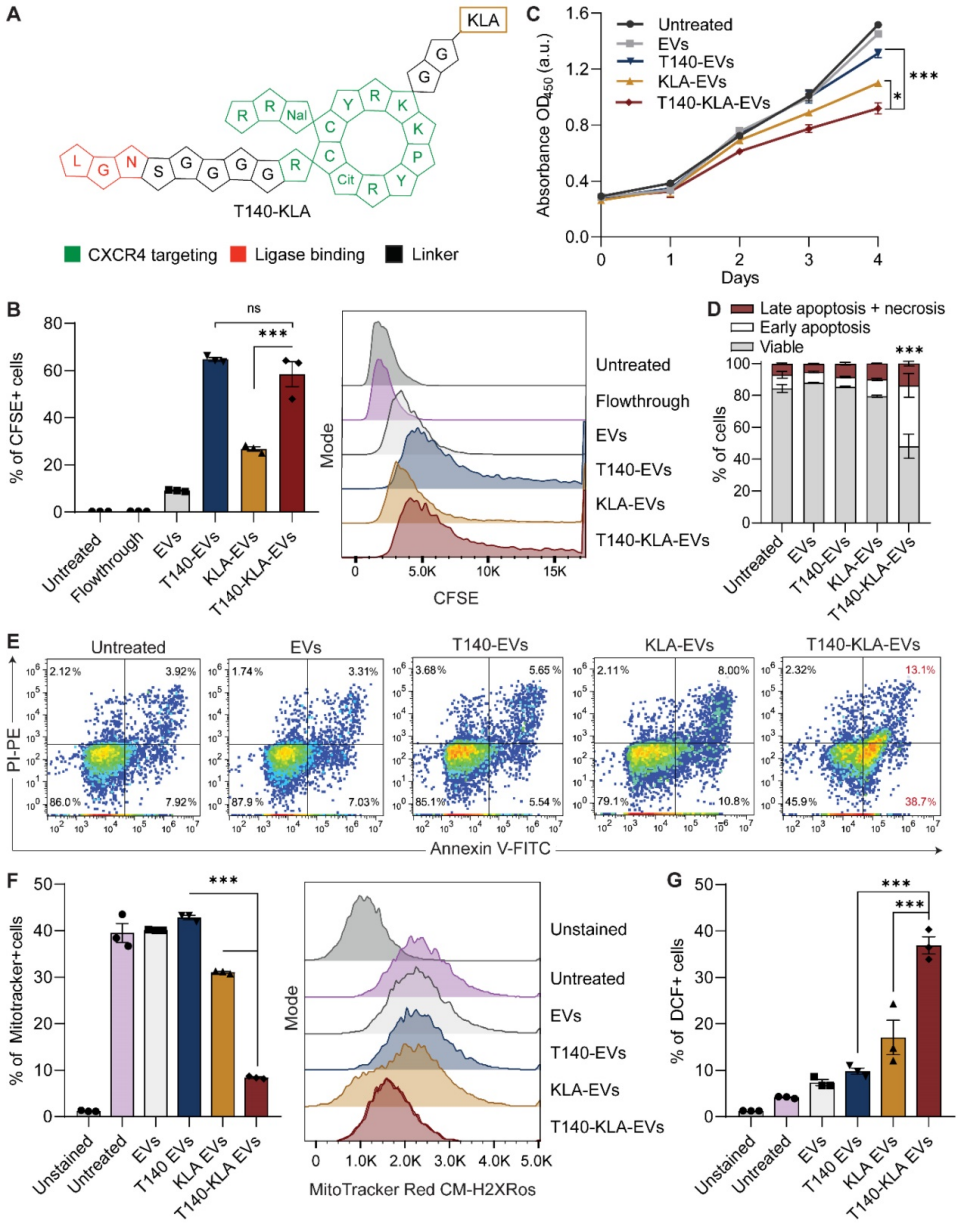
** Targeted delivery of a bifunctional apoptotic peptide leads to suppression of cancer cell survival. (A)** Design of the bifunctional T140-KLA peptide in which KLA is a 14 -amino-acid proapoptotic peptide (full sequence is shown in Table S1).** (B)** *In vitro* uptake of T140-, KLA- and T140-KLA-conjugated RBCEVs by MOLM13 cells, determined using flow cytometry analysis of CFSE that labels RBCEVs. **(C)** Proliferation of MOLM13 cells treated with unmodified RBCEVs, or peptide-conjugated RBCEVs over a period of 4 days. Proliferation was quantified using CCK8 assay and represented as absorbance at 450 nm (a.u., arbitrary unit). **(D)** Percentage of apoptotic cells 4 days after treatment with unmodified or peptide-conjugated RBCEVs, determined based on Annexin V and PI staining. Annexin V^+^ PI^-^ cells are considered early apoptotic cells. Annexin V^+^ PI^+^ cells are considered late apoptotic cells. Annexin V^-^ PI^+^ cells are considered necrotic cells. **(E)** Representative annexin V/PI flow cytometry plots for each treatment from **D**. **(F)** Mitochondrial membrane potential of MOLM13 cells after each EV treatment, quantified using flow cytometric analysis of MitoTracker Red CM-H2XRos, a membrane-potential dependent mitochondria stain. **(G)** Comparison of total cellular ROS following treatment with uncoated or peptide coated EVs obtained via flow cytometric analysis of DCF. Graphs **B**, **C**, **D**, **F** and **G** represent data from 3 biological replicates prepared from RBCEVs from independent donors. The graphs present the mean ± SEM. Student's one-tailed t-test: ns - not significant, *P < 0.05, ***P < 0.001.

KLA is a potent pro-apoptotic peptide that induces mitochondrial-dependent apoptosis once internalized into cells 17, but its poor permeability across the eukaryotic plasma membrane and indiscriminate cellular toxicity limit its therapeutic application. To improve the specificity of KLA-induced toxicity, we conjugated its C-terminal to the K residue on the T140 peptide via a GG linker (Figure 3A). This bifunctional T140-KLA peptide was subsequently ligated onto the RBCEV surface as described above. T140-KLA-EVs are a three-component system where each part plays a key irreplaceable function: the T140 peptide provides CXCR4-specific targeting activity, the KLA peptide induces a pro-apoptotic effect and EVs improve the cellular uptake, circulation kinetics and biocompatibility of the peptide components in the body.

We first verified if the addition of the KLA sequence impeded CXCR4-specific docking of the T140 peptide ligated RBCEVs. As judged by flow cytometry, KLA conjugation did not affect the targeting ability of the T140-KLA-EVs in MOLM13 cells, with T140-KLA-EVs showing comparable uptake to T140-EVs (Figure 3B). Afterwards, we evaluated the impact of T140-KLA-EVs treatment on MOLM13 cells. A time-course treatment revealed that T140-KLA-EVs significantly reduced the overall growth of MOLM13 cells after three days of incubation, suggesting that the KLA domain exerted its pro-apoptotic effects (Figure 3C). KLA-EVs also showed a slight decrease in cellular proliferation, but less effectively than T140-KLA-EVs. The induction of apoptosis in MOLM13 cells was subsequently quantified using annexin V and PI staining for each EV treatment. After 4 days of treatment, the total apoptotic rate (early apoptosis, late apoptosis and necrosis) in untreated cells and cells treated with unmodified EVs or T140-EVs was similar (~12%) and significantly lower than that of EVs coated with KLA (~21%) (Figure 3D-E). Of note, the T140-KLA-EVs showed the highest apoptotic effect as evidenced by over 50% of the cells stained positive for annexin V, indicating high phosphatidylserine exposure. To test the specificity of the apoptotic effect mediated by T140-KLA-EVs, we repeated the annexin V/PI assay in CXCR4-negative AsPC-1 cells. Following 4 days of treatment with similar doses of each EV treatment, we noted that both KLA and T140-KLA-EVs induced very low levels of apoptosis (~16%) compared to control and untreated cells (~9%) (Figure S4B-C). Importantly, there was no significant difference between T140-KLA-EV and KLA-EV treatments. Taken together, these data indicate that while the KLA-domain can induce low levels of non-discriminate toxicity towards cells, the addition of the T140 domain results in potent CXCR4-directed toxicity via the increased delivery of these EVs to CXCR4-expressing cells.

We subsequently investigated how the T140-KLA-EVs exerted their apoptotic effects by quantifying the mitochondrial membrane potential (MMP) and reactive oxidative species (ROS) production in cells incubated with different EV treatments. After 24 hours, MOLM13 cells incubated with T140-KLA-EVs showed significantly lower MitoTracker Red CM-H2XRos accumulation and elevated levels of ROS activity (Figure 3F-G, S4D). KLA-EVs showed much less significant effects while the other EV treatments had no detectable effects on either MitoTracker accumulation or ROS activity. Thus, it can be concluded that T140-KLA-EVs negatively impacted the MMP of treated cells, leading to a significant enhancement in the production of ROS, resulting in increased apoptosis.

While our experiments thus far have shown that T140-KLA-EVs can induce CXCR4-targeted cell death in a very specific manner, there is also a concern that these EVs could induce toxicity to normal body cells such as CD4-positive T-cells that also have high CXCR4 expression. Isolation of CD4-positive T cells from human PBMCs followed by CXCR4-staining revealed that human primary CD4-positive T cells do in fact express high, albeit heterogenous levels of CXCR4 (S5A-B). To further investigate the potential for off-target toxicity to such cells, we conducted an *in vitro* uptake assay to assess how T140 peptide ligation affected EV uptake by CD4-positive T cells. Our data revealed that T140 peptide conjugation resulted in a slight increase in EV uptake as compared to unconjugated EVs (Figure S5C). However, no significant difference was observed between control peptide ligated and T140-EVs. Upon closer examination, we determined that T cells showed significantly lower levels of EV uptake as opposed to the AML MOLM13 cells used in this study, which may attribute to the low efficacy of targeted delivery to T cells, despite the presence of CXCR4. We also conducted annexin V/PI apoptosis assay on T cells to determine if T140-KLA-EVs induced increased cell death. Notably, treatment with T140-KLA-EVs did not increase the total apoptotic ratio of CD4-positive T cells compared to KLA-EVs (Figure S5D-E). We did however; observe a slight increase in apoptosis in both KLA and T140-KLA treated T cells. However, CCK8 analysis of activated CD4-positive T cells revealed that treatment with either KLA-EVs or T140-KLA-EVs over a period of 4 days did not result in significant decreases in viability as compared to untreated or control EV treated cells (Figure S5F). Taken together with the fact that EVs do not remain in circulation for prolonged periods of time, these results suggest that T140-KLA-EV treatments are unlikely to lead to targeted T cell toxicity upon systemic administration.

### EVs are efficiently conjugated with antibodies via a linker peptide and the streptavidin-biotin system

Despite the efficacy of enzymatic ligation demonstrated above, this approach is feasible only for low molecular weight molecules such as peptides that can be easily modified to include a ligation motif. We have previously developed a two-step ligation method to conjugate EVs with nanobodies via a linker peptide 8. However, this method required the addition of a ligase-binding motif to the nanobody and the use of high concentrations of the nanobody to maintain a high rate of enzymatic ligation. Moreover, not all cancer-associated surface markers have validated low molecular weight targeting molecules or nanobodies that display the high affinity and specificity offered by monoclonal antibodies. The addition of a ligase binding motif to monoclonal antibodies is complex and time consuming, making the ligation of these large proteins unfeasible.

To extend the versatility of the surface functionalization approach and achieve more efficient targeting, we utilized the streptavidin-biotin system to conjugate larger and more complex molecules such as nanobodies and monoclonal antibodies on the surface of EVs. The biotin functional group was incorporated onto EVs first via enzymatic ligation of a biotinylated peptide (B-TL5) followed by sequential incubation with the tetrameric protein streptavidin (SA) and a biotinylated targeting molecule of choice (Figure S6A). Monoclonal antibodies were commercially obtained pre-biotinylated while nanobodies were purified and biotinylated in-house. Given the four distinct biotin binding pockets per molecule of streptavidin, it is capable of further amplifying the copy number of antibodies conjugated per EV. Moreover, the streptavidin-biotin system serves as a potent linker with a dissociation constant (K_d_) in the order of ~10^-14^ mol/L, one of the strongest non-covalent interactions in nature 18. As a result, this method provides a stable conjugation between the antibody and EVs while also preserving the biocompatible conjugation methods used throughout this project.

**Figure 4:**
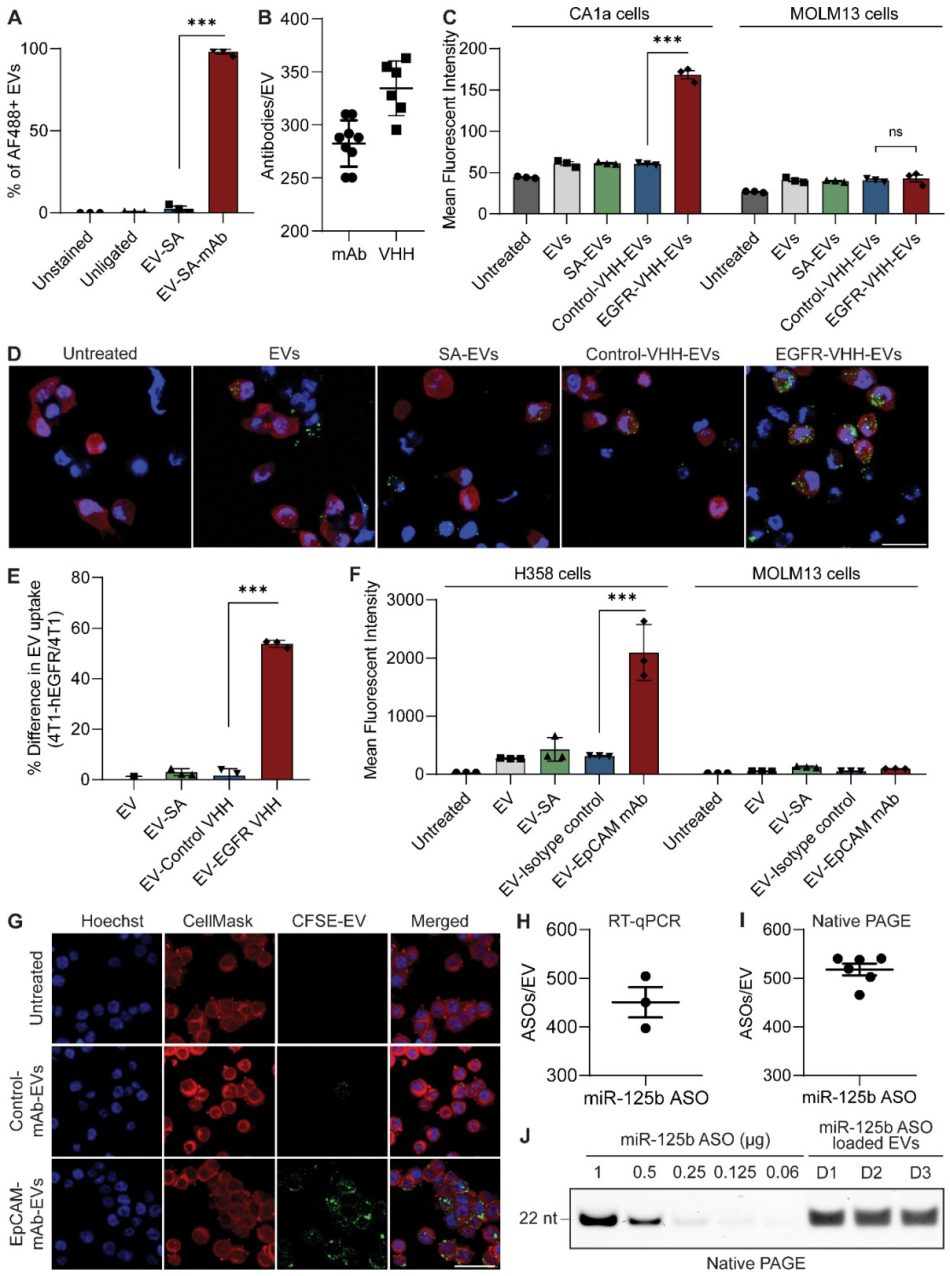
** Antibody conjugated RBCEVs show increased accumulation in target cells.** RBCEVs were conjugated with biotinylated monoclonal antibodies or nanobodies via a biotinylated linker peptide and streptavidin**. (A)** Single EV flow cytometric analysis of monoclonal antibody conjugation on RBCEVs using the streptavidin-mediated conjugation method. **(B)** Copy number of isotype control monoclonal antibody (mAb) and nanobodies (EGFR VHH) per RBCEV quantified using ELISA by comparison to a standard curve of antibodies/nanobodies (n = 6-9 replicates). **(C)** Flow cytometry analysis of CFSE in EGFR-positive CA1a cells or EGFR-negative MOLM13 cells treated with CFSE-labeled RBCEVs coated with control or EGFR-targeting nanobodies. **(D)** Representative immunofluorescent images of EV uptake in the co-culture of 4T1 and 4T1-tdTomato-hEGFR cells incubated with different RBCEV treatments. RBCEVs were tracked using CFSE (green), tdTomato was shown in red while nuclei were co-stained with Hoechst (blue). Scale bar is 20 µm.** (E)** Percentage difference in RBCEV uptake between 4T1-tdTomato-hEGFR cells and parental 4T1 cells, expressed as a fraction of mean CFSE intensity for each RBCEV treatment. **(F)** Uptake of EpCAM-targeted or control CFSE-labelled RBCEVs by EpCAM-positive H358 cells or EpCAM-negative MOLM13 cells. **(G)** Representative immunofluorescent images of EpCAM-targeting and non-targeting RCBEV uptake by H358 cells as in **(F)**. RBCEV uptake was observed using CFSE (green). CellMask was used to label the cell membrane (red) and the nucleus was visualized using Hoechst (blue). Scale bar is 50 µm.** (H)** RT-qPCR quantification of miR-125b ASOs loaded per EV obtained via comparison of the total RNA extract from miR-125b ASO-loaded EVs to a standard curve of miR-125b ASO (n = 3 biological replicates).** (I)** Quantification of miR-125b ASOs loaded per individual EV obtained via native PAGE analysis of miR-125b ASO-loaded RBCEVs electrophoresed alongside a serial dilution of unloaded miR-125b ASO (n = 6 biological replicates)**. (J)** Representative native PAGE analysis used to assess the loading efficiency of ASOs into EVs. Each EV lane denotes a separate biological replicate prepared using EVs from 3 blood donors (D1-D3). Graphs **A, C, E** and **F** represent data from 3 biological replicates prepared from RBCEVs from independent donors. For quantification of RNA per EV in **H** & **I**, NTA was used to obtain the number of input EVs, thereby providing an estimate of ASO copy number per EV. The graphs present the mean ± SEM. Student's one-tailed t-test: ns - not significant, ***P < 0.001.

Single-EV flow cytometric analysis of isotype monoclonal antibody-conjugated EVs revealed that RBCEVs could be efficiently conjugated with monoclonal antibodies using the B-TL5 peptide via the streptavidin method (Figure 4A). Moreover, the flow cytometry profiles revealed a homogenous conjugation of antibodies on the EV surface as visualized by the shift in fluorescence of the entire EV population upon monoclonal antibody conjugation (Figure S7A). While our previous data on peptide ligation had indicated that the B-T140 peptide led to a higher copy number than B-TL5, we decided to utilize the B-TL5 peptide as the primary linker peptide for streptavidin-mediated antibody conjugation to avoid the selective binding of EVs to CXCR4 while using antibodies targeting other receptors. Moreover, we hypothesized that the streptavidin itself could amplify the copy number via its multiple free binding sites, compensating for the decrease in copy number via using B-TL5. Quantification of the copy number of monoclonal antibodies per EV using ELISA revealed that each RBCEV was conjugated to ~282 antibodies on average when used in conjunction with B-TL5 and streptavidin (Figure 4B). Nanobodies which were significantly smaller were conjugated at slightly higher copy number, averaging ~334 copies per EV (Figure 4B). Of note, we did not detect any crosslinking of EVs caused by streptavidin, presumably due to the large excess of streptavidin and antibodies utilized at each stage of conjugation (Figure S3D, G).

### Monoclonal antibody and nanobody functionalized RBCEVs accumulate preferentially in target cells

After confirming that RBCEVs could be efficiently conjugated with intact monoclonal antibodies and nanobodies, we sought to verify the functionality of antibody-functionalized EVs. To this end, we tested if the EV-immobilized antibodies still retained their binding affinity and specificity using two models of surface antigen targeting: EGFR targeting in EGFR-positive MCF10CA1a (CA1a) breast cancer cells or 4T1-tdTomato-hEGFR mouse breast cancer cells using an anti-human EGFR biparatopic camelid-derived single domain antibody (VHH), and EpCAM targeting in EpCAM-positive NCI-H358 lung cancer cells using a commercially obtained anti-human EpCAM monoclonal antibody (Figure S6B-G). We used a cell association assay to determine if control or targeting antibody-conjugated EVs could bind to cells expressing the corresponding receptor. Following incubation of targeting or non-targeting RBCEVs with cells at 4 °C, cell-bound RBCEVs were detected by staining with an antibody against glycophorin A (GPA), a specific surface marker for human RBCs and consequently RBCEVs. In each cell model, we observed that targeting antibody-conjugated EVs displayed high affinity for target cells expressing the corresponding receptor, while control antibody-conjugated EVs or unconjugated EVs showed no affinity for target cells (Figure S7B-C). Furthermore, neither the targeted or control EVs showed affinity for a negative cell line, MOLM13, which had low or no expression of both target receptors, thereby highlighting the specific nature of targeting. Following confirmation of the ability of targeting EVs to home in and bind to target cells specifically, all subsequent *in vitro* experiments were conducted at 37 °C in an effort to measure EV uptake, which is a more clinically relevant readout, where the capability of targeted EVs to specifically accumulate inside target cells is assessed.

*In vitro* uptake of EGFR VHH or control VHH-conjugated RBCEVs was assessed in EGFR-positive CA1a cells and EGFR-negative MOLM13 cells separately, yielding a similar pattern to that observed in the cell association assay (Figure 4C, S8A). EV uptake was monitored using flow cytometric analysis of cellular CFSE fluorescence from CFSE-labelled EVs as detailed in the methods section. While non-targeted RBCEVs were taken up at low levels by CA1a cells, in the presence of EGFR targeting, EV uptake was increased significantly as displayed by the two-fold increase in mean intracellular CFSE signals. To further validate the targeting ability of EGFR VHH-conjugated EVs, we created a mouse 4T1 cell line stably co-expressing tdTomato and human EGFR (4T1-tdTomato-hEGFR) (Figure S6C, G), and co-cultured these cells with the parental 4T1 cells. Equal numbers of 4T1-tdTomato-hEGFR and parental 4T1 cells were pre-seeded and incubated with 5 μg EGFR targeting or control CFSE-labelled RBCEVs for 2 hours followed by flow cytometry and immunofluorescent analysis. The 4T1-tdTomato-hEGFR cells were distinguished from 4T1 cells based on tdTomato expression (Figure S6G). Figure 4D shows representative immunofluorescent images from the co-culture uptake of cells incubated with each EV treatment, confirming the increased intracellular accumulation of EGFR targeting EVs in the tdTomato-hEGFR double-positive 4T1 cells (visualized in red), but not in the tdTomato-hEGFR double-negative parental 4T1 cells (visualized as the cells with only nuclear staining in blue). Figure 4E displays flow cytometric analysis data to present the data quantitatively as the percentage difference in mean CFSE fluorescent intensity for each EV treatment in 4T1-tdTomato-hEGFR as compared to the hEGFR-negative 4T1 cells, corroborating the immunofluorescence data in Figure 4D (Figure S8B). These data suggest that targeting persists even in the presence of more complex microenvironments consisting of non-target cells.

Targeting EpCAM with EpCAM mAb-conjugated RBCEVs also resulted in increased uptake of RBCEVs in EpCAM-positive H358 cells, but not in EpCAM-negative MOLM13 cells (Figure 4F, S8C). Isotype control mAb-conjugated RBCEVs or streptavidin-conjugated RBCEVs had no effect on the basal level of RBCEV uptake in either cell line. This data was also corroborated via immunofluorescent imaging, which revealed increased uptake in the EpCAM-mAb-EV treatment as evidenced by the increased distribution of intracellular CFSE visualized as bright green speckles inside the H358 cells (Figure 4G, S8D). We also verified that the EVs were inside the cells and not merely stuck to the surface using confocal microscopy to obtain Z-stacks at 100 × magnification, thereby visualizing EVs within the cells (Figure S8E). Taken together, these data indicate that antibody-conjugated EVs can be utilized for tumor cell-specific delivery of EV-encapsulated payloads.

### Antibody-mediated targeting promotes the specific delivery of EV-encapsulated RNA to target cells

After verifying the ability of antibody-conjugated RBCEVs to specifically accumulate in target cancer cells, we sought to use this platform to deliver encapsulated RNA cargos specifically to target cells. RBCEVs were loaded with nucleic acid cargos using REG1 or Exo-Fect and enzymatically surface functionalized prior to incubation with cells (Figure S9A). Subsequent quantification of RNA loading was carried out via RT-qPCR. In brief, total RNA was extracted from miR-125b ASO-loaded RBCEVs using TRIzol. A TaqMan miRNA assay designed against the miR-125b ASO sequence was used to quantify the ASOs in the extracted RNA alongside a standard curve of miR-125b ASO. Comparison of Ct values revealed that each EV contained on average 450 miR-125b ASOs (Figure 4H). This was further corroborated using native PAGE, comparing EV-loaded RNA to a standard curve of the input RNA which gave an estimate of ~518 molecules of RNA per EV, corresponding to a loading efficiency of approximately 87.5% (Figure 4I-J). We also conducted native PAGE analysis of unloaded and ASO-loaded EVs side-by-side to demonstrate that the visualized bands were contributed solely by the loaded ASOs and not by endogenous RNAs that maybe present in RBCEVs (Figure S9B). The lack of RNA bands in the RBCEV only control is attributed to very low levels of endogenous RNA that is beyond the detection limit of the GelRed nucleic acid stain used for visualization. Furthermore, single flow cytometric analysis of FAM ASO-loaded EVs revealed that the loaded EVs showed a homogenous population, suggesting consistent loading across all the EVs (Figure S9C). This further supports the quantification data obtained using RT-qPCR and native PAGE where the total ASOs present in a large number of EVs was divided by the number of EVs to obtain the number of ASOs per EV.

**Figure 5:**
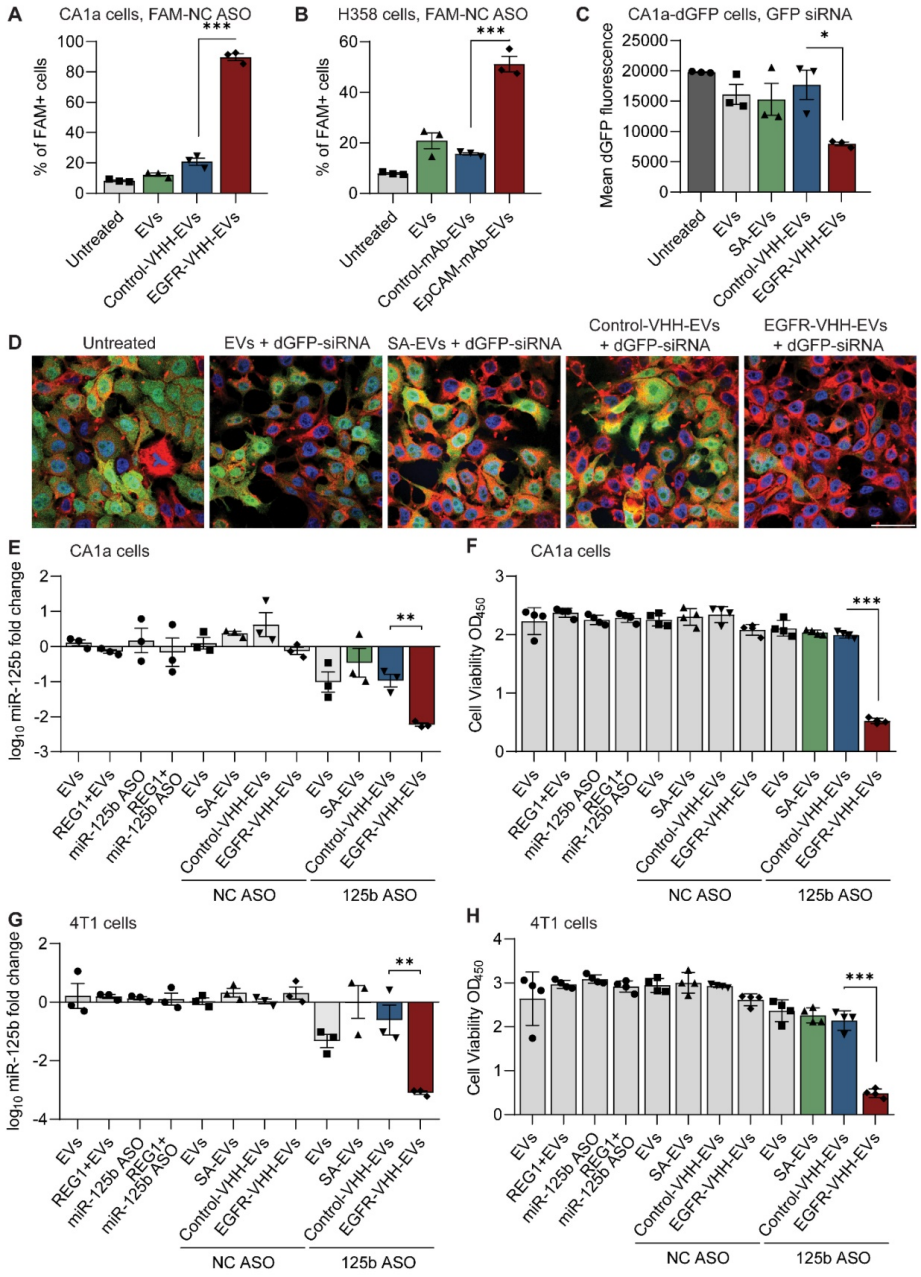
** Targeted delivery of DNA/RNA payloads to target cells using antibody functionalized RBCEVs. (A)** Flow cytometry analysis reflecting the delivery of a FAM-conjugated non-targeting ASO (FAM-NC-ASO) to CA1a cells by EGFR-targeting or non-targeting RBCEVs.** (B)** Flow cytometry analysis demonstrating the delivery of FAM-ASO to H358 cells using EpCAM-mAb-conjugated RBCEVs or non-targeting RBCEVs. **(C)** Mean dGFP fluorescence of CA1a-dGFP cells determined using flow cytometry following incubation with EGFR-targeted or non-targeted RBCEVs loaded with GFP-siRNA. **(D)** Representative immunofluorescent images of CA1a-dGFP cells treated with dGFP-siRNA-loaded RBCEVs. Cells were stained with CellMask dye (red) while dGFP expression is shown in green. Scale bar is 20 µm.** (E)** Knockdown of miR-125b in CA1a cells using EVs loaded with NC-ASO or 125b-ASO loaded RBCEVs, with or without EGFR targeting. miR-125b was quantified using TaqMan RT-qPCR, normalized to U6b RNA and presented as average log_10_ fold change relative to the untreated control. **(F)** Effect of NC-ASO or 125b-ASO loaded RBCEVs on the viability of CA1a cells, assessed using CCK8 assay. **(G)** miR-125b knockdown (normalized to snoRNA234) and **(H)** viability assays in 4T1-hEGFR cells performed similarly as in **E** & **F**. Graphs **A**,** B**, **C**, **E**, **F**, **G** and **H** represent data from 3 biological replicates prepared from RBCEVs from independent donors. The graphs present the mean ± SEM. Student's one-tailed t-test: *P < 0.05, **P < 0.01, ***P < 0.001.

We initially validated the ability of RBCEVs to increase accumulation of RNA payloads inside cells using a FAM-conjugated negative control (NC) ASO. Flow cytometry and immunofluorescent analysis demonstrated that EGFR nanobody-functionalized EVs increased the accumulation of FAM ASO in EGFR-positive CA1a cells (Figure 5A, S10A). We also repeated this experiment using EpCAM monoclonal antibody-conjugated RBCEVs targeting EpCAM-positive H358 cells, showing a similar increase in encapsulated FAM-ASO delivery only in the presence of antibody-mediated EV targeting (Figure 5B, S10B). Importantly, confocal imaging at high magnification of CA1a cells treated with FAM ASO-loaded EGFR-targeted EVs revealed that the FAM signal was present inside the cell (Figure S10C). This was followed up by a functional readout, where GFP siRNA-loaded, EGFR-targeted RBCEVs were utilized to achieve specific knockdown of destabilized GFP (dGFP) in CA1a-dGFP cells. While non-targeted EVs decreased the expression of dGFP by ~20%, in the presence of EGFR targeting, the dGFP level was found to be well below 60% of the untreated control (Figure 5C). This was also corroborated by immunofluorescent imaging, demonstrating almost complete knockdown of dGFP in the majority of cells in the EGFR-targeted treatment after 4 hours of incubation (Figure 5D).

Lastly, we loaded RBCEVs with ASOs against miR-125b, a tumorigenic microRNA upregulated in many cancer cell lines including breast cancer CA1a and 4T1 cells 19. Dose response assays were used to obtain a sub-optimal dose that would induce partial knockdown of miR-125b and a ~20% decrease in cell viability (Figure S10D-E). A dose of 2.5 µg EVs was decided upon for subsequent knockdown assays while 5 µg EVs were used for the viability assays in both cell lines. Evaluation of knockdown of miR-125b in CA1a cells using targeted and non-targeted RBCEVs demonstrated that RBCEVs coated with EGFR-targeting VHH resulted in a ~10-times greater fold change in miR-125b levels (Figure 5E). Equimolar amounts of naked ASO added directly to cells or ASO-REG1 complexes treated similarly to EVs showed no significant increase in knockdown. Moreover, EGFR targeted RBCEVs loaded with miR-125b ASO significantly decreased the viability of CA1a cells to ~20% compared to the untreated control (Figure 5F), an effect attributed to the increased suppression of miR-125b levels in this treatment. A similar trend in miR-125b knockdown and cell viability was also seen in 4T1-hEGFR cells (Figure 5G-H). These data indicate that tumor cell-targeted RBCEVs loaded with miR-125b ASO could be used for therapeutic purposes.

### EGFR-targeted delivery of miR-125b ASO-loaded RBCEVs efficiently slows down cancer progression

**Figure 6:**
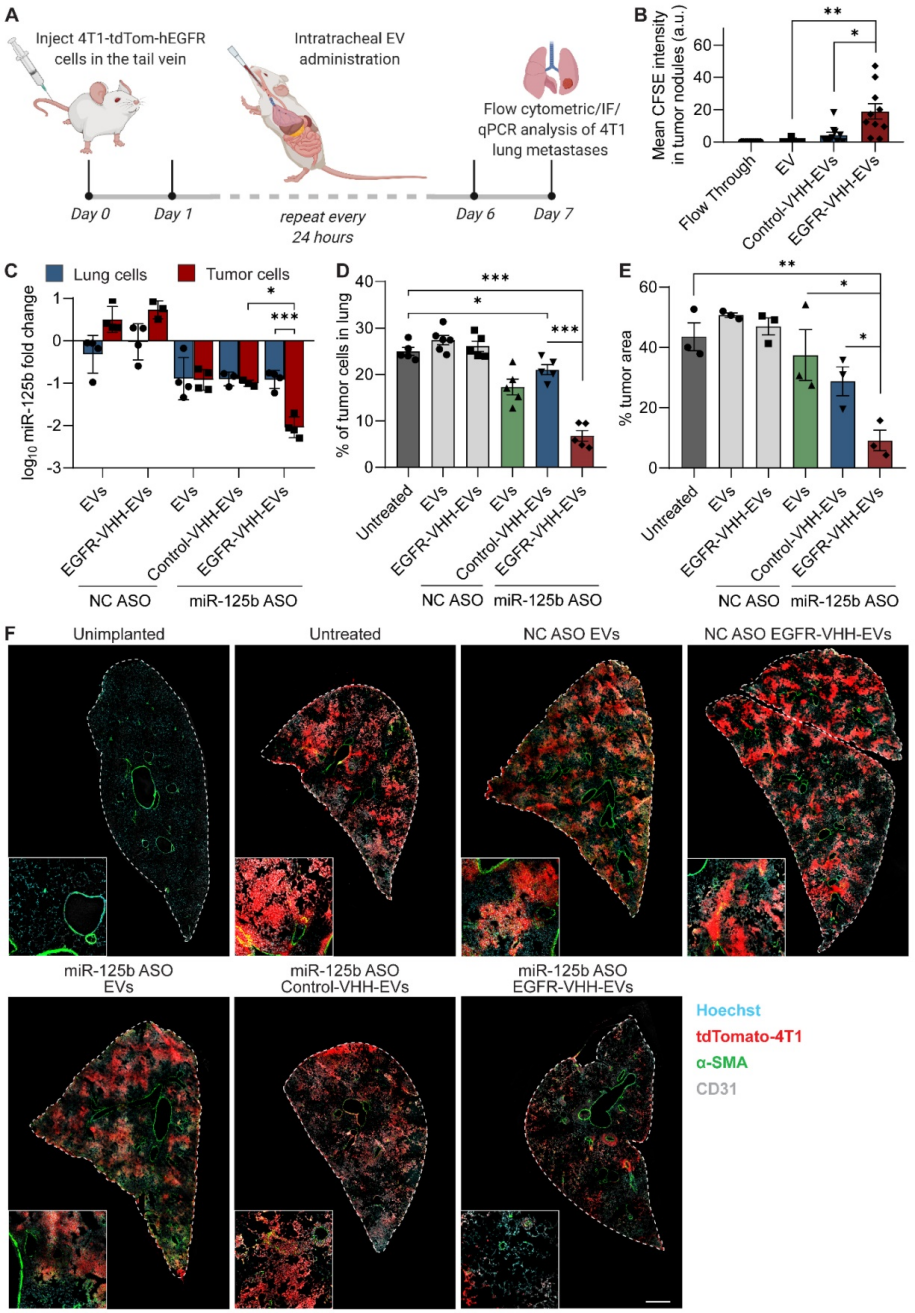
** EGFR-targeted miR-125b ASO loaded EVs efficiently suppress tumor progression. (A)** Overview of the *in vivo* study used to evaluate the efficacy of antibody-conjugated EVs for targeted delivery of RNA therapeutics. **(B)** Evaluation of EV uptake by tumor cells in the lung for EGFR-targeted and non-targeted CFSE-labeled EVs determined based on the colocalization of CFSE and tdTomato signals using immunofluorescent imaging of lung sections. Each data point corresponds to a single tile scan of the lung acquired at random. **(C)** Relative miR-125b knockdown in mouse lung cells and tumor cells following administration with different EV treatments. RNA levels were normalized to snoRNA (n = 3-4 mice). **(C)** Percentage of tumor cells in the lung at the endpoint of the experiment for each treatment condition, obtained using flow cytometric analysis of tdTomato and hEGFR double positive cells in lung homogenates (n = 5-6 mice). **(D)** Percentage of lung area occupied by tumor tissue, obtained via quantification of tdTomato fluorescence of intact lung sections. **(E)** Representative immunofluorescent images of intact lung sections from each treatment condition stained for α-SMA and CD31. Tumor tissue was identified by the tdTomato fluorescence and shown in red. Scale bar, 1 mm. Inset shows selected areas at 4 × magnification. The graphs present the mean ± SEM. Student's one-tailed t-test: *P < 0.05, **P < 0.01, ***P < 0.001.

To demonstrate the target specificity of antibody conjugated EVs in delivering RNA payloads, we developed an hEGFR-positive lung metastatic breast cancer model by injecting 4T1-tdTomato-hEGFR cells systemically into NSG-SGM3 mice with the intention of administering the EV treatment intratracheally (Figure 6A). Initial uptake studies using intratracheally administered CFSE-labelled EVs in tumor bearing mice revealed that EGFR-targeted EVs showed significantly higher levels of uptake by tumor cells as compared to untargeted EVs (Figure 6B, S11A). We also investigated the ability of intratracheal EV administration to deliver EVs to the entirety of the lung by analyzing transverse lung sections of mice administered with a single dose of CFSE-labelled EVs. Analysis of tiles scans obtained from separate sections spaced approximately 0.5 mm apart was used to plot a graph of percentage lung coverage by EVs in each section. The data revealed that the majority of EVs were distributed within 2-5 mm form the top of the lung with lung coverage between 20-60% (Figure S11B). The upper and lower extremities had relatively lower coverage, though clear EV signals were still detected, albeit at lower abundance.

Once we had verified the ability of EVs to be efficiently distributed throughout the lung and to accumulate preferentially in hEGFR-positive tumor cells, we administered a single dose of NC ASO or miR-125b ASO-loaded EVs with or without EGFR targeting to tumor-bearing mice 5 days post-inoculation. After 36 hours, the mice were sacrificed and lung homogenates sorted using a cell sorter to separate equal numbers of tumor and non-tumor cells (Figure S11C). RT-qPCR of these cells reflected a similar trend to that observed in the uptake assay, with EGFR-targeted miR-125b ASO-loaded EVs resulting in significantly higher knockdown only in tumor cells, but not in lung cells (Figure 6C). Non-targeted miR-125b ASO loaded EVs showed moderate knockdown in both lung and tumor cells while NC ASO-loaded EVs showed no clear knockdown effect.

We followed this up with a treatment regimen consisting of daily intratracheal administration of 400 µg miR-125b or NC ASO-loaded EVs with or without EGFR targeting for 6 days. The mice were sacrificed on day 7 when the untreated group showed signs of weakness and the lungs were excised and homogenized. Flow cytometric analysis of lung cells from each treatment revealed that EGFR-VHH-EVs loaded with miR-125b ASO demonstrated the best tumor suppressive effects, averaging at ~5% of tumor cells in the lung compared to 25-30% in the untreated and NC ASO-EV treated mice and 15-20% in untargeted miR-125b ASO-EV treated mice (Figure 6D). This pattern was also reflected in the tdTomato fluorescence quantification of intact lung sections from each treatment (Figure 6E-F). Notably, upon closer examination of lung sections, we noticed that tumor nodules in the EGFR-targeted miR-125b-EV treatment were significantly smaller and did not show high levels of colocalization with CD31, a well-established marker for blood vessel density in tumor tissue (Figure 6F). This is in contrast to the other treatment conditions that displayed markedly larger pulmonary tumor nodules (tdTomato fluorescence shown in red) with strong colocalization with elevated levels of CD31 (shown in grey). Collectively, this data demonstrates the translatability of targeted EVs for achieving improved therapeutic outcomes with RNA-based cancer therapies.

### CXCR4-targeted pro-apoptotic EVs suppress leukemia progression and improve the survival of AML xenografted mice

**Figure 7:**
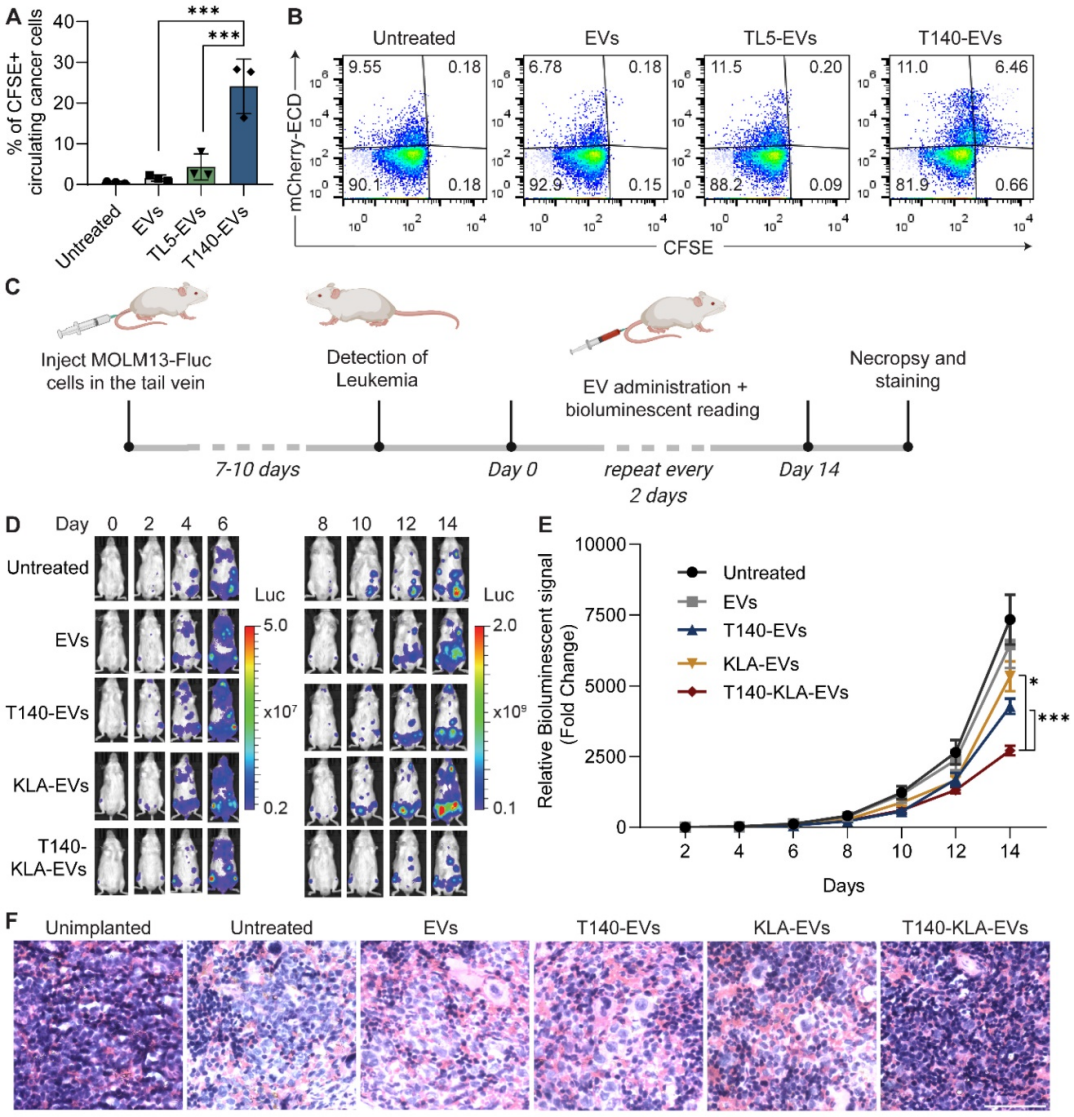
** T140-KLA-EVs suppress leukemia development and improves survival of AML xenografted mice. (A)** Percentage of circulating leukemia MOLM13-mCherry cells that uptake EVs following a single systemically administered dose of EVs. EV uptake was tracked using CFSE and analyzed using flow cytometry (n = 3 mice).** (B)** Representative flow cytometry plots illustrating the uptake of CFSE-labelled EVs by circulating cells. mCherry expression is used to distinguish leukemia MOLM13 cells from mouse PBMC. **(C)** Outline of the animal study for verifying the efficacy of KLA-T140-EVs in leukemia suppression. **(D)** Representative bioluminescent images of NSG mice implanted with CXCR4-positive luciferase-expressing MOLM13 cancer cells during a course of systemic RBCEV treatments. Colors indicate bioluminescent signals (photon/s) in 2 scales (the images are divided into 2 groups, day 0-6 and day 8-14 from the treatment start date, to avoid oversaturation of signals). **(E)** Average bioluminescent signals quantified in the mice during the development of leukemia (photons/s), normalized by the signals at the start of the treatments, and presented as mean ± SEM. (n = 7 mice).** (F)** Representative images of H&E staining of spleen sections from leukemic mice for each treatment condition. Scale bar, 50 µm. Student's one-tailed t-test (A) and Two-way ANOVA test (E): *P < 0.05, ***P < 0.001.

After verifying the *in vitro* cytotoxic effects of EVs with T140-KLA peptide conjugation, we sought to determine if this approach was translatable *in vivo* by assessing relative EV uptake, leukemia progression, off-target toxicity and overall survival of leukemia xenografted mice. Here, we used an AML mouse model in which NSG mice were injected with luciferase and mCherry labelled MOLM13 cells. Leukemia development was monitored using bioluminescent imaging of the mice. To investigate the uptake of CXCR4-targeted EVs by circulating leukemia cells, late-stage AML mice were injected systemically with targeted or control CFSE-labelled EVs. Peripheral blood cells were analyzed by flow cytometry 4 hours post-administration, thus revealing the presence of a distinct population of mCherry-positive MOLM13 cells in circulation (Figure S12A). Subsequent analysis of this subpopulation of cells revealed that the conjugation of RBCEVs with the T140 peptide significantly increased the percentage of CFSE-positive tumor cells from ~2% in the control groups to ~25% in the T140-EVs treatment (Figure 7A). Of note, administration of T140-EVs led to an appreciable increase of CFSE fluorescence in the entire mCherry-positive population of leukemia cells while non-leukemic cells in the same treatment did not show any detectable levels of EV uptake, further exemplifying the specific nature of T140-mediated EV targeting (Figure 7B).

This data also encouraged the use of bifunctional peptide coated EVs for the treatment of AML-xenografted mice. After 1 week of leukemia inoculation, when the leukemic bioluminescent signals became visible, the mice were treated with RBCEVs coated with T140, KLA or T140-KLA peptides followed by bioluminescent measurement every 2 days (Figure 7C). Figure 7D shows representative bioluminescent images of mice at 2-day intervals for each treatment condition, while figure 7E plots the relative bioluminescence signals of all mice normalized to the initial reading before the first treatment. The data demonstrates that by day 14, the leukemic bioluminescence in mice treated with T140-KLA-EVs decreased significantly compared to the other groups. H&E staining of spleen sections, the most common site for leukemia metastasis 20, confirmed that the treatment with T140-KLA-EVs reduced the infiltration of leukemia cells in the spleen as evidenced by the lower number of leukemic cells (identified by the characteristic large nuclei and large nuclear-cytoplasmic ratio) (Figure 7F, S12B). Furthermore, the mice treated with T140-KLA-EVs had a significantly higher symptom-free survival rate as compared to other treatment conditions (Figure S12C).

In addition, given the potential for off-target toxicity induced by the pro-apoptotic domain of the T140-KLA peptide, we wanted to exclude the possibility that T140-KLA-EVs could induce toxicity in organs such as the liver that show high characteristic EV uptake upon systemic treatment. To this end, we collected the sera from AML-xenografted mice following a single systemic dose of EVs (40 mg/kg) with or without the bifunctional peptide coating and analyzed the level of bilirubin, ALP, ALT and AST. We did not observe any significant differences in the biochemistry panels between each group, indicating that the T140-KLA-EVs did not induce hepatoxicity in leukemia xenografted mice (Figure S12D). Taken together, these results indicate that T140-KLA-EVs are safe and effective in treating leukemia *in vivo* by potentiating the apoptotic effects of the KLA peptide specifically in CXCR4-positive leukemia cells, thereby slowing the progression of the disease and improving the overall survival.

### Surface-modified RBCEVs are non-toxic and non-inflammatory

**Figure 8:**
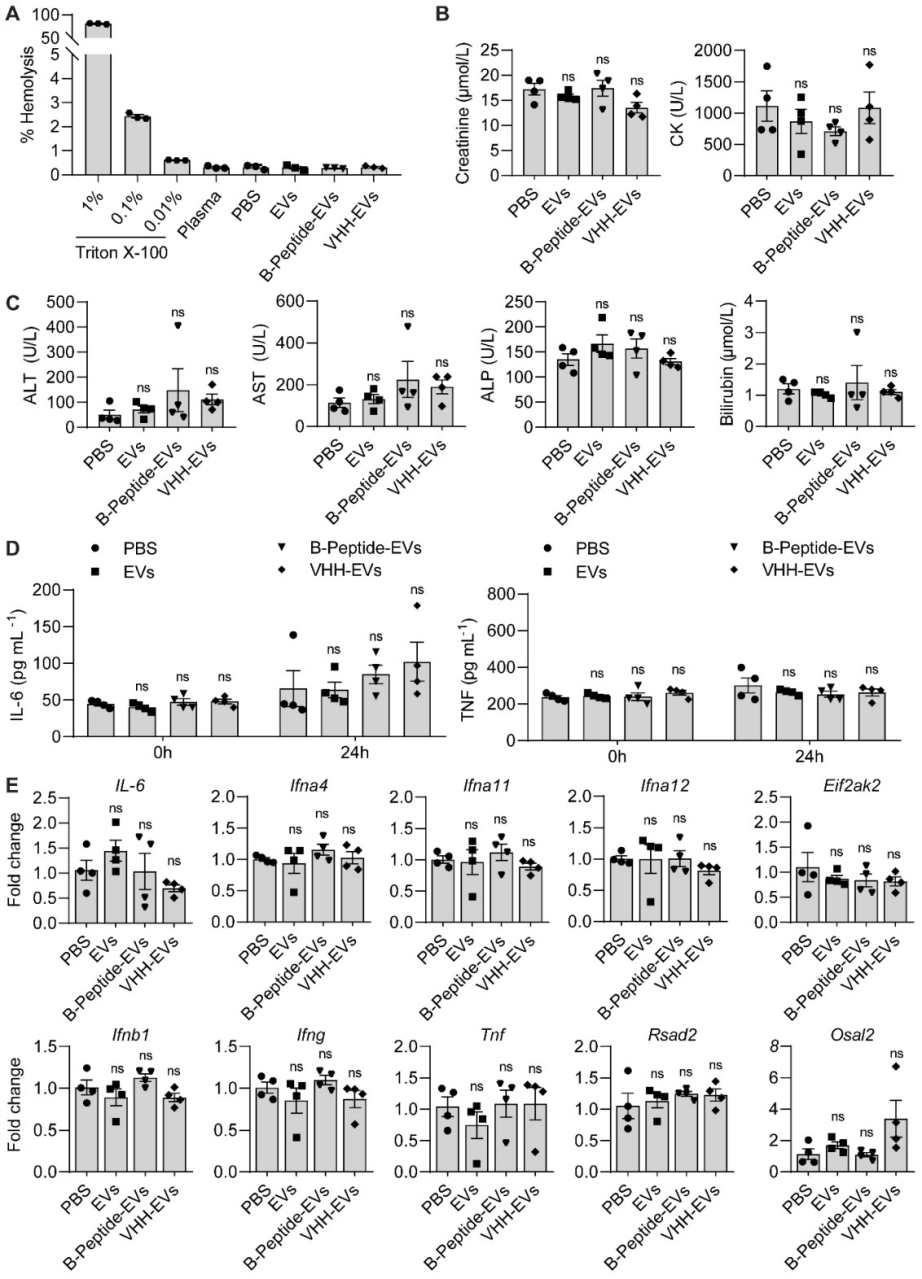
** Surface engineered RBCEVs maintain a non-toxic, safe and biocompatible profile. (A)** Hemolysis assay to assess hemolytic potential of RBCEVs with or without OaAEP1-mediated surface coating. RBCEVs were conjugated with a control peptide or nanobody and incubated with fresh human whole blood at 37°C for 1 hour. Intact RBCs were removed and the supernatant was assessed for hemoglobin content. Percentage of hemolysis is shown relative to a positive control where RBCs were completely lysed with ACK buffer. Incubation with 0.01-1% Triton X-100 is included for reference. **(B)** Creatinine and CK levels in the serum of C57BL/6 mice 24 hours after an i.v. injection of 40 mg/kg uncoated RBCEVs or the same dose of control peptide-coated or VHH-coated RBCEVs or an equal volume of PBS. **(C)** AST/ALT/ALP/bilirubin levels in the serum of RBCEV-treated mice as described in **B**.** (D)** IL-6 and TNF level in the serum of C57BL/6 mice 0-24 hours after an i.v. injection of RBCEVs as described in **B**. **(E)** RT-qPCR analysis of genes related to immune responses in the liver 24 hours after the injection of RBCEVs as described in **B**. Graphs B, C, D and E display data from 4 mice per group, presented as mean ± SEM. Student's one-tailed t-test: ns - not significant.

To investigate the potential safety of surface-modified EVs for therapeutic applications, we initially tested the compatibility of surface-modified human RBCEVs with human blood, particularly the hemolytic potential of RBCEVs. RBCEVs were incubated at the highest concentration intended to be used for intravenous administration (0.5 mg/mL) with fresh human whole blood at 37 °C for 1 hour. Neither unmodified nor peptide/antibody-conjugated RBCEVs showed any sign of hemolytic potential after incubation in fresh human whole blood, while in the positive control, hemolysis was clearly observed after 0.1-1% Triton X-100 treatments (Figure 8A). Subsequently, we analyzed the general toxicity and immunogenicity levels following intravenous injection of biotinylated peptide or VHH coated RBCEVs into C57BL/6 mice (at a dosage of 40 mg/kg, equal to that of the therapeutic dose used in this study). We did not observe any significant differences among treatment groups when we evaluated the level of creatinine and creatine kinase (CK), key markers for kidney function and muscle damage respectively (Figure 8B). We also tested RBCEVs for liver toxicity, given the propensity of RBCEVs to accumulate at high levels in the liver. Remarkably, there was no difference across several liver toxicity parameters including ALT, AST, ALP and total bilirubin levels between sham injections and each EV treatment, indicating that EVs do not induce liver toxicity or adversely affect liver function (Figure 8C).

Next, we investigated the *in vivo* immune-stimulatory potential of RBCEVs in immunocompetent C57BL/6 mice. Serum and tissues were collected and analyzed 24 hours following RBCEV administration as reported in the materials and methods section. As shown in Figure 8D, the concentrations of IL-6 and tumor necrosis factor (TNF) in the serum of mice administered with different EV treatments were similar to that of the mice injected with an equal volume of PBS, indicating the absence of an acute immune response upon EV administration. This data was further supported by qPCR quantifying the mRNA expression of immune response-related genes in the liver, where no difference was observed between the EV treatments and the sham PBS injection (Figure 8E). Taken together, these data suggest that the systemic administration of peptide/antibody coated EVs do not induce detectable levels of *in vivo* toxicity or acute inflammation in immunocompetent mice, supporting their safe use for therapeutic delivery.

## Discussion

Advances in nanomedicine have enabled effective delivery of chemotherapeutics and RNA drugs, leading to the approval and clinical application of several nanoparticle-formulated products including DOXIL® (doxorubicin in liposome), Abraxane® (albumin-bound paclitaxel), Onivyde® (irinotecan in liposome), Onpattro™ (siRNAs in lipid nanoparticles) and most recently several mRNA-based COVID-19 vaccines 21-23. Drug formulation with nanoparticles significantly enhances the drug solubilization, stability, and penetration of cellular barriers 1. Moreover, the next generation of nanomedicine is developing cutting-edge technologies to further improve the specificity and safety of drug delivery. EVs as nature's delivery vehicles have recently emerged as a new class of nanomedicines with great biocompatibility. As EVs are naturally taken up by many cell types and deliver various biomolecules for intercellular signaling, they have great potential to deliver pharmaceutical drugs efficiently and safely. However, the ability of EVs to be taken up readily by most cell types poses a drawback in terms of achieving delivery specificity. When administered systemically, a large fraction of EVs tend to accumulate in the liver due to phagocytosis by Kupffer cells 24. The rest of the EVs are distributed to various tissues depending on the natural homing tendency of EVs, which is based on their parental cells and method of isolation 25. However, the accumulation of systemically administered EVs, even cancer cell-derived EVs, to solid-cancer tumors and their uptake by cancer cells is very low, limiting the potential clinical application of EV-based drug delivery methods in systemic cancer treatment. Hence, an approach for surface modification of EVs with tumor-targeting moieties is desirable for improving the efficacy of EV-based drug delivery for anti-cancer therapy.

As mentioned previously, existing methods for surface modifications of EVs such as genetic manipulation, affinity conjugation, lipid insertion and chemical treatments have various shortcomings in terms of safety, stability and integrity. Here, we have demonstrated the utility of enzymatically engineered EVs that show potent antigen-specific targeting abilities, capable of homing in on target cells and delivering therapeutic cargoes with high efficiency and specificity. The resulting EVs possessed targeting molecules at copy numbers well in excess of most existing methods. More importantly, via the streptavidin-biotin system, we demonstrated the ability to efficiently conjugate intact monoclonal antibodies on the EV surface, an approach that could in theory be extended to most other biotinylated molecules, regardless of its chemical composition or size.

We also establish that this approach is relevant for the functional delivery of peptide and RNA-based therapeutics for the *in vivo* treatment of cancer models, either via the enzymatic ligation of peptide drugs or the exogenous loading of RNAs. These engineered EVs were able to improve the biodistribution of encapsulated and ligated drugs, significantly slowing down cancer progression. In theory, this approach could be expanded for the delivery of other nucleic acid-based therapeutics, peptides or biotinylated molecules that could be conjugated using the approach outlined in this study. Taking into consideration the biocompatibility, stability, versatility and efficiency presented by enzymatically modified EVs, this surface functionalization approach presents a novel breakthrough in creating functionally translatable EV-based drug delivery vectors.

The entirety of this study utilizes RBC-derived EVs though we have previously shown the translatability of the enzymatic approach to EVs from other cell sources 8. This is because RBCs provide a scalable, cost-effective and safe source of EVs, devoid of any risk of genomic contamination as has been demonstrated in some depth in previous studies 6. We have also provided an in-depth analysis of the immunogenicity and safety of these engineered RBCEVs, assessing them on their potential for hemolysis, liver damage or generation of an immune response. Despite the propensity of RBCEVs to naturally accumulate in the liver, we detected no signs of toxicity or adverse effects on the liver after administration of RBCEVs, which may be explained by the ability of Kupffer cells to efficiently break down any endocytosed material, a function for which they are naturally suited. Moreover, none of the RBCEV treatments showed any indication of hemolytic potential or induction of acute inflammation. Taken together, this indicates that enzymatically engineered RBCEVs are a suitable, and possibly safer alternative to currently used drug delivery vectors.

The production of RBCEVs, peptides, antibodies and OaAEP1 ligase is simple, scalable and cost effective. O-negative packed RBCs from any blood bank can be used for production of universal RBCEVs, where each unit of RBCs can generate sufficient RBCEVs for a few treatments of human patients 6. Meanwhile, *E. coli* can be used for large-scale low-cost production of OaAEP1 ligase and nanobodies, which can subsequently be conveniently purified and modified as required without affecting their function 8. Peptides can be chemically synthesized with ease using established protocols at low cost. No expense is required for mammalian cell culture, viral production or lipid synthesis which are usually the costly factors associated with AAV or nanoparticle production. Hence, these targeted RBCEVs can be produced on a large scale at competitive cost.

However, more investigation is desirable to assess the long-term adaptive immune response to engineered RBCEVs, an aspect that is not investigated in our study due to the complication of interspecies treatment of mice with human RBC-derived EVs. The production of EVs from mouse RBCs is challenging due to the small blood volume of mice and extensive lysis of mouse RBCs upon calcium ionophore treatment. Hence, further immunogenicity and toxicity assays should be done in larger animals such as dogs and monkeys which can provide more substantial volumes of blood and RBCs with more similar characteristics to humans 26. Moreover, despite the effectiveness of the streptavidin-mediated approach for conjugating large protein molecules at high copy number with unparalleled stability and efficiency, there are concerns regarding the induction of an immune response to streptavidin. While our acute immunogenicity tests showed no sign of inflammation on the short term, it is possible that following long term treatment, some patients may develop anti-streptavidin antibodies which could decrease the efficacy of subsequent treatments. As such, it may be necessary to use hypoimmunogenic muteins of streptavidin that retain both immune evasion and retention of function 18.

## Supplementary Material

Supplementary figures and table.

## Abbreviations

EVs: extracellular vesicles
RBCEVs: red blood cell-derived extracellular vesicles
OaAEP1: Oldenlandia affinis asparaginyl endopeptidase
mAb: monoclonal antibody
CXCR4: chemokine receptor type 4
EGFR: epidermal growth factor receptor
EpCAM: epithelial cell adhesion molecule
ASO: antisense oligonucleotide
PBS: phosphate buffered saline
RBCs: red blood cells
CFSE: carboxyfluorescein diacetate succinimidyl ester
SEC: size exclusion chromatography
DiR: 1,1'-dioctadecyl-3,3,3',3'-tetramethylindotricarbocyanine iodide
Hb: Hemoglobin
VHH: variable heavy homodimers
IPTG: Isopropyl β-D-1-thiogalactopyranoside
PMSF: phenylmethylsulphonyl fluoride sulfonyl fluoride
RIPA: radioimmunoprecipitation assay
PE: phycoerythrin
GPA: glycophorin A
VSSC: violet side scatter
EM: electron microscopy
PI: propidium iodide
MMP: mitochondrial membrane potential
ROS: reactive oxygen species
ROIs: regions of interest
NSG: NOD scid gamma
AML: acute myeloid leukemia
IVIS: *in vivo* imaging system
NC: negative control
a.u.: arbitrary units
