# Supplementary material for: Surface-engineered extracellular vesicles for targeted delivery of therapeutic RNAs and peptides for cancer therapy
Source: Theranostics. 2022 Apr 11;12(7):3288–315. doi: 10.7150/thno.68667 (PMC9065173; doi:10.7150/thno.68667)
Supplement: Supplementary file 1 — Supplementary figures and table. [file thnov12p3288s1.pdf]

Supplementary data

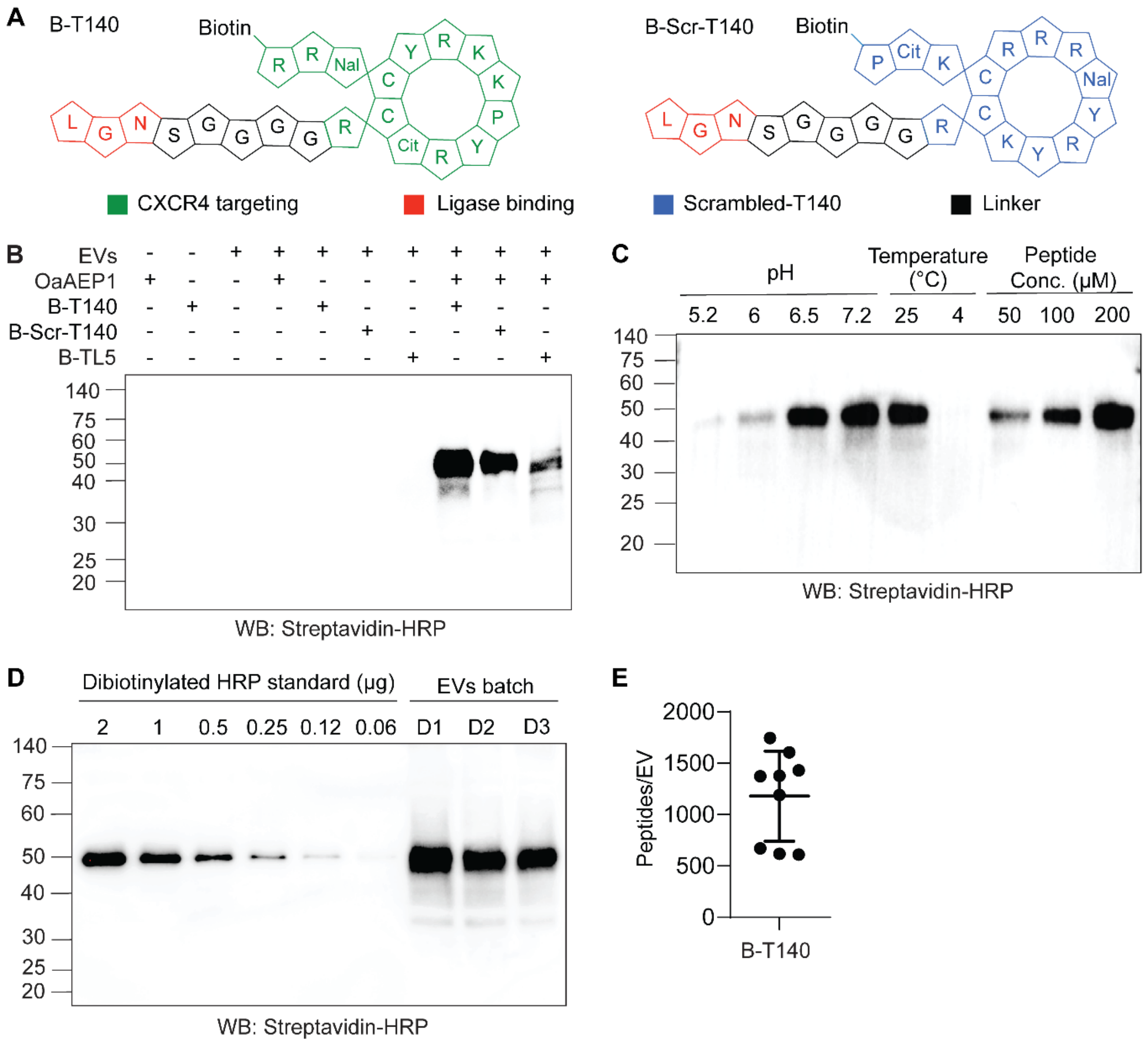

Figure S1| Verification and quantification of enzymatic peptide-EV ligation. (A) Design of the B-T140 and B-Scr-T140 peptides. Cysteine 4 and cysteine 13 are conjugated through a disulfide bridge; Nal: L-3-(2-naphthyl)-alanine; Cit: L-citrulline. **(B)** Western blot analysis of biotin on B-T140, Scr-B-T140 and B-TL5 peptides ligated on RBCEVs. **(C)** Optimization of ligation conditions: B-T140 peptide was ligated to RBCEVs under different conditions and analyzed for ligation efficiency using a biotin western blot. **(D)** Western blot of B-T140 peptide ligated onto RBCEVs from 3 different donors (D1-3) compared to a serial dilution of dibiotinylated HRP. The blot was probed with streptavidin-HRP. **(E)** Copy number of B-T140 peptide per EV based on western blot analysis (n = 9 replicates).

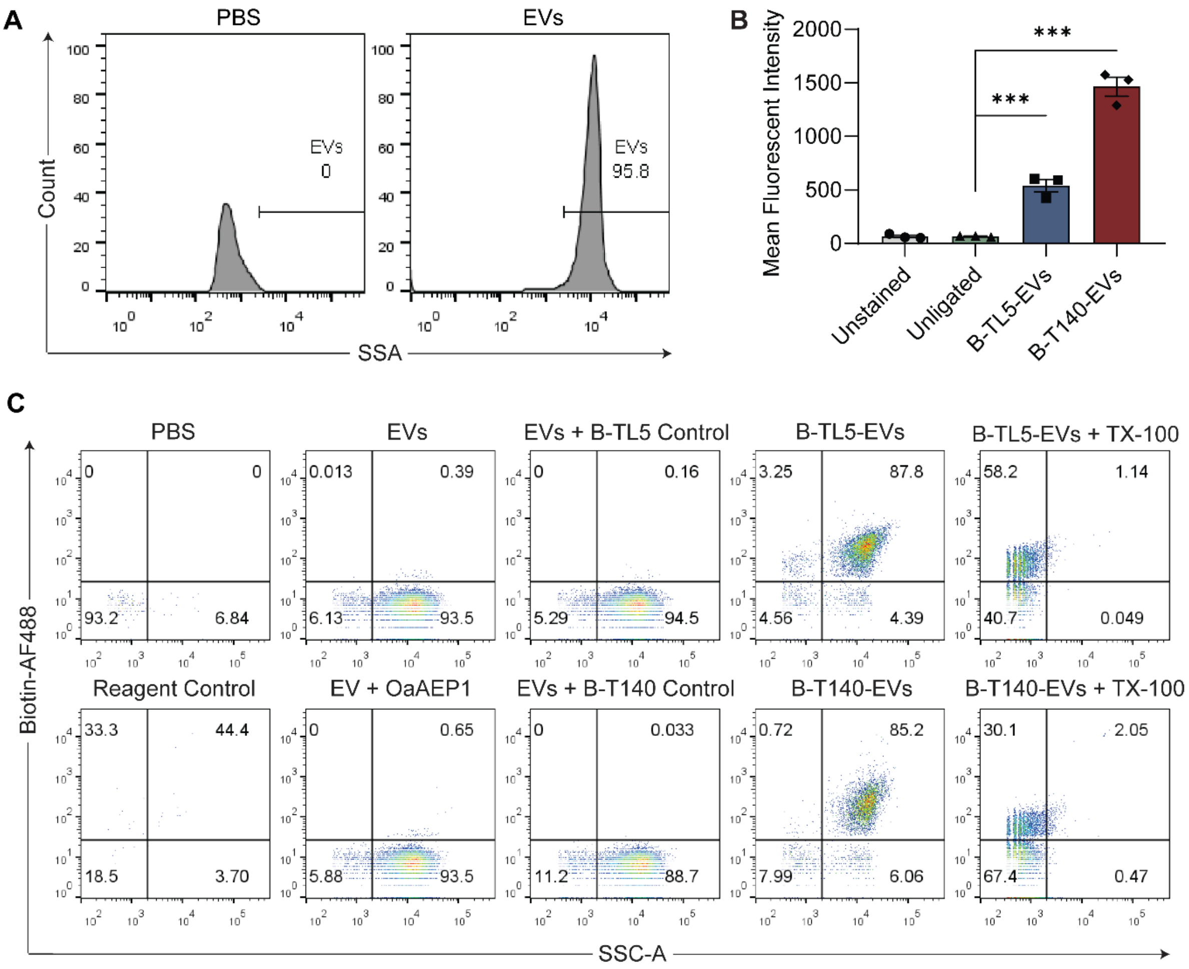

Figure S2| Characterization of enzymatic peptide-EV ligation. (A) Gating strategy used for distinguishing RBCEVs from the background for single EV flow cytometry. **(B)** Mean fluorescent intensity of the biotinylated peptide signal on B-TL5/B-T140-ligated or unligated EVs. **(C)** Representative flow cytometry plots of RBCEV-peptide ligation, showing all events. EVs were gated from background based on SSC-A intensity and the relative biotin signal measured following staining with streptavidin-AF488. All controls as per MIFlowCyt guidelines including PBS only, reagents control and detergent controls have been included to demonstrate the clear analysis of single EVs. The graph presents the mean ± SEM from samples prepared from RBCEVs derived from 3 distinct donors. Student's one-tailed t-test: ***$P < 0.001$

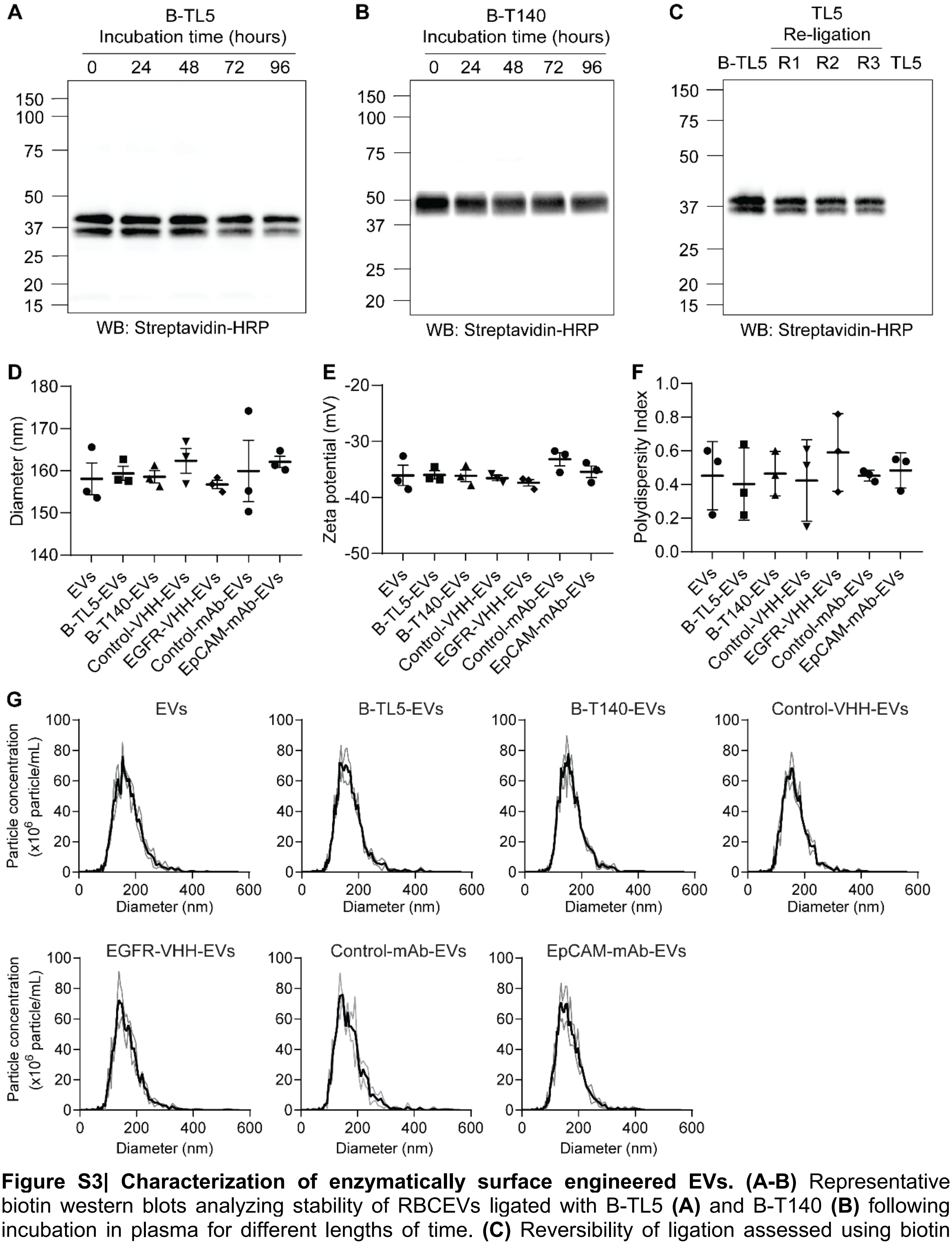

Figure S3| Characterization of enzymatically surface engineered EVs. (A-B) Representative biotin western blots analyzing stability of RBCEVs ligated with B-TL5 **(A)** and B-T140 **(B)** following incubation in plasma for different lengths of time. **(C)** Reversibility of ligation assessed using biotin western blot to monitor the percentage of ligated B-TL5 peptide remaining following 3 rounds of repeated (R1-3) ligation with a non-biotinylated peptide (TL5). **(D)** Hydrodynamic diameter, **(E)** zeta potential and **(F)** polydispersity index (PDI) of RBCEVs coated with either peptides or antibodies, obtained using a Litesizer™ 500 or a Particle Metrix ZetaView® particle analyzer. For figures **D-F**, n = 3 replicates, where RBCEVs for each replicate were obtained from a different donor. **(G)** Size distribution of RBCEVs unmodified or conjugated with peptides, nanobodies or monoclonal antibodies, determined using a Particle Metrix ZetaView® particle analyzer.

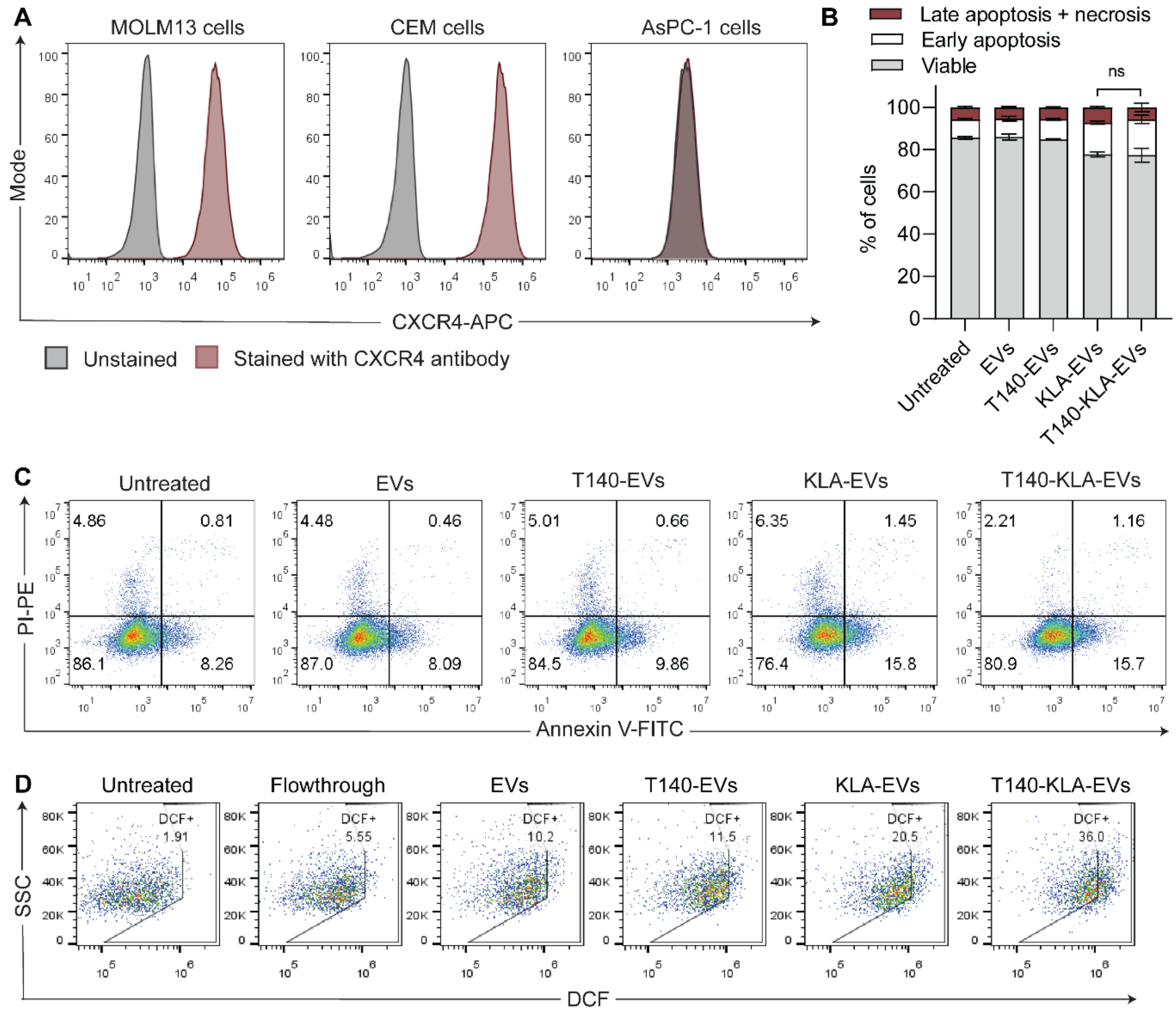

Figure S4| CXCR4-targeting T140 peptide ligation induces CXCR4-specific EV uptake. (A) Flow cytometry analysis of CXCR4 expression in leukemia MOLM13 cells, leukemia CEM cells and pancreatic adenocarcinoma AsPC-1 cells determined using an APC-conjugated anti-CXCR4 antibody. **(B)** Apoptotic ratios of CXCR4-negative cells following treatment for 96 hours with T140-KLA-EVs, KLA-EVs, T140-EVs or control EVs determined using annexin V/PI staining. **(C)** Representative flow cytometry plots of Annexin V/PI staining in AsPC-1 cells for each treatment condition in **B. (D)** Representative flow cytometry plots showing dichlorofluorescein (DCF) fluorescence in MOLM13 cells treated with uncoated, T140, KLA or T140-KLA coated EVs. Following EV treatment, cells were incubated with 1 μM DCF for 30 minutes at room temperature before being analyzed by flow cytometry. Student's one-tailed t-test: ns = not significant

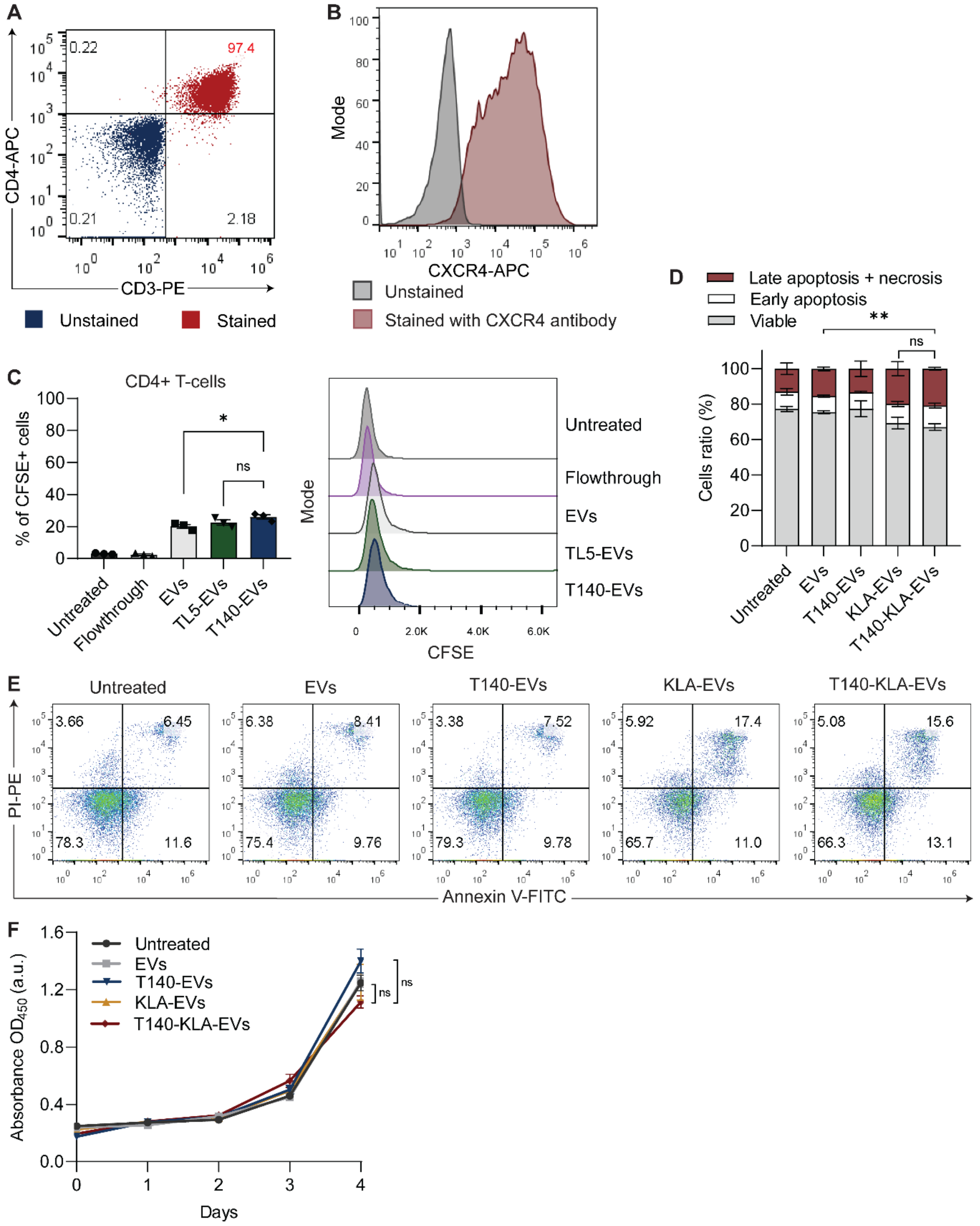

Figure S5| Effect of CXCR4-targeting T140-KLA-EVs on CD4-positive T cells. (A) Flow cytometry analysis of CD3 and CD4 in CD4-positive cells following isolation using magnetic separation to deplete CD14 monocytes and enrich CD4-positive T cells. Cells from the unstained sample are shown superimposed on the same plot in blue while the stained cells are shown in red. Percentages in each quadrant are shown for the stained sample. **(B)** CXCR4 expression in T cells from **A.** **(C)** In vitro uptake assay of CFSE-labelled T140-EVs or control EVs by T-cells prepared as in **A**. **(D)** Percentage of apoptotic cells 4 days after treatment with T140-KLA-EVs, KLA-EVs, T140-EVs or control EVs assessed using Annexin V/PI staining. Annexin V+ PI- cells are considered early apoptotic cells. Annexin V+ PI+ cells are considered late apoptotic cells. Annexin V- PI+ cells are considered necrotic cells. **(E)** Representative annexin V/PI flow cytometry plots for each treatment from **D**. **(F)** Assessment of the effect of each EV treatment from **D** on activated CD4-positive T cell viability and proliferation determined using CCK8 assay. Student's one-tailed t-test: ns = not significant, *P<0.05, **P<0.01

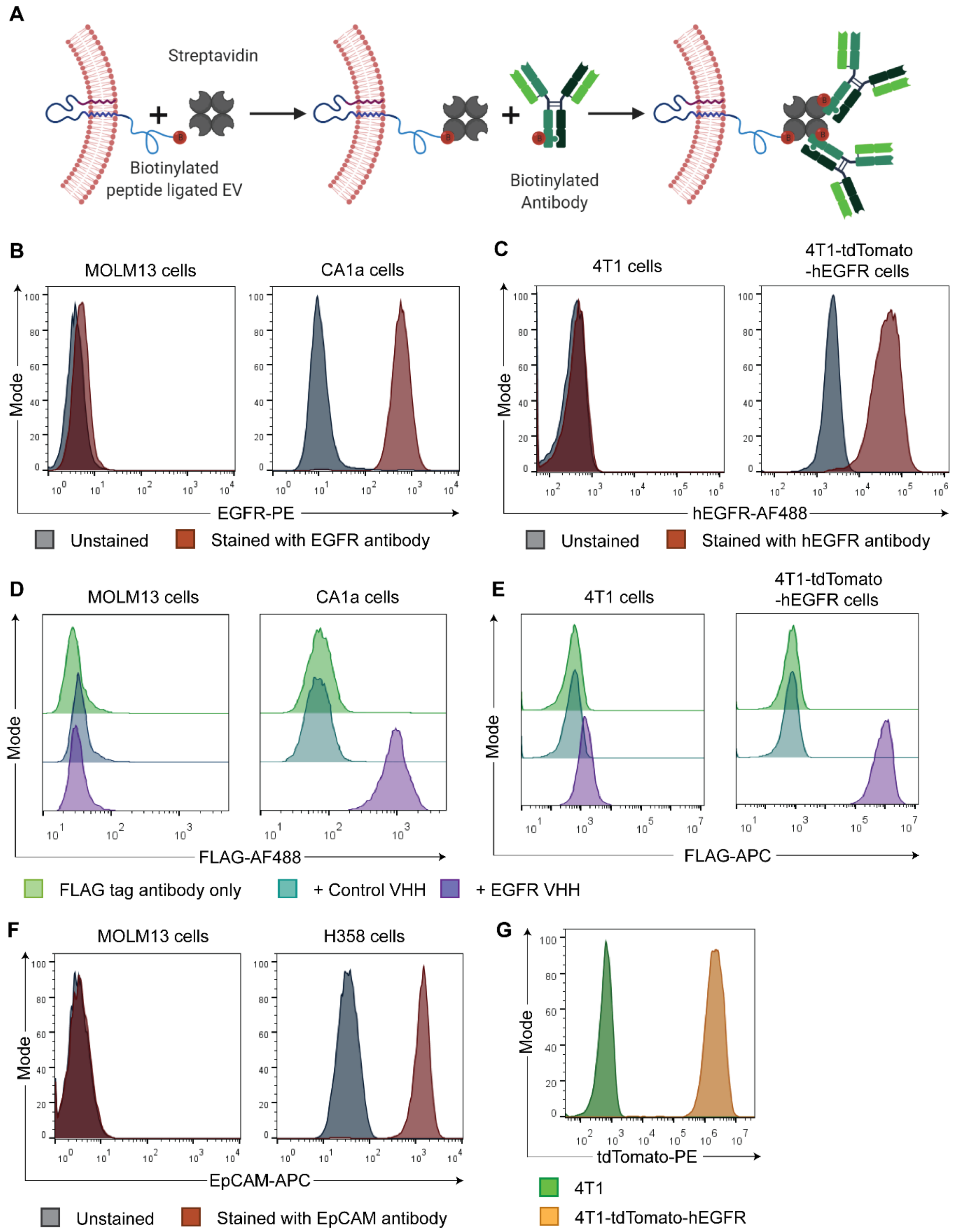

Figure S6| Engineering EVs with antibodies to target cancer-specific markers. (A) Schematic of the conjugation of peptide-ligated RBCEVs with biotinylated antibodies using streptavidin. RBCEVs were first ligated with a biotinylated peptide, followed by an incubation with streptavidin and a biotinylated nanobody (VHH) or monoclonal antibody (mAb) of choice. **(B)** Flow cytometric analysis of EGFR expression in breast cancer CA1a cells and leukemia MOLM13 cells analyzed using a PE-conjugated anti-EGFR antibody. **(C)** Flow cytometry analysis of human EGFR (hEGFR) in mouse breast cancer 4T1 cells with and without tdTomato and hEGFR overexpression using a human EGFR specific antibody conjugated with AF488. **(D)** Binding of the biotinylated EGFR VHH to different cell lines, detected using an AF488 antibody that recognized the FLAG tag on the VHH. **(E)** Binding of the biotinylated EGFR VHH to 4T1 and 4T1-hEGFR cells. **(F)** Expression of EpCAM in human lung H358 cells and leukemia MOLM13 cells. **(G)** tdTomato expression in 4T1 parental cells and 4T1-tdTomato-hEGFR cells.

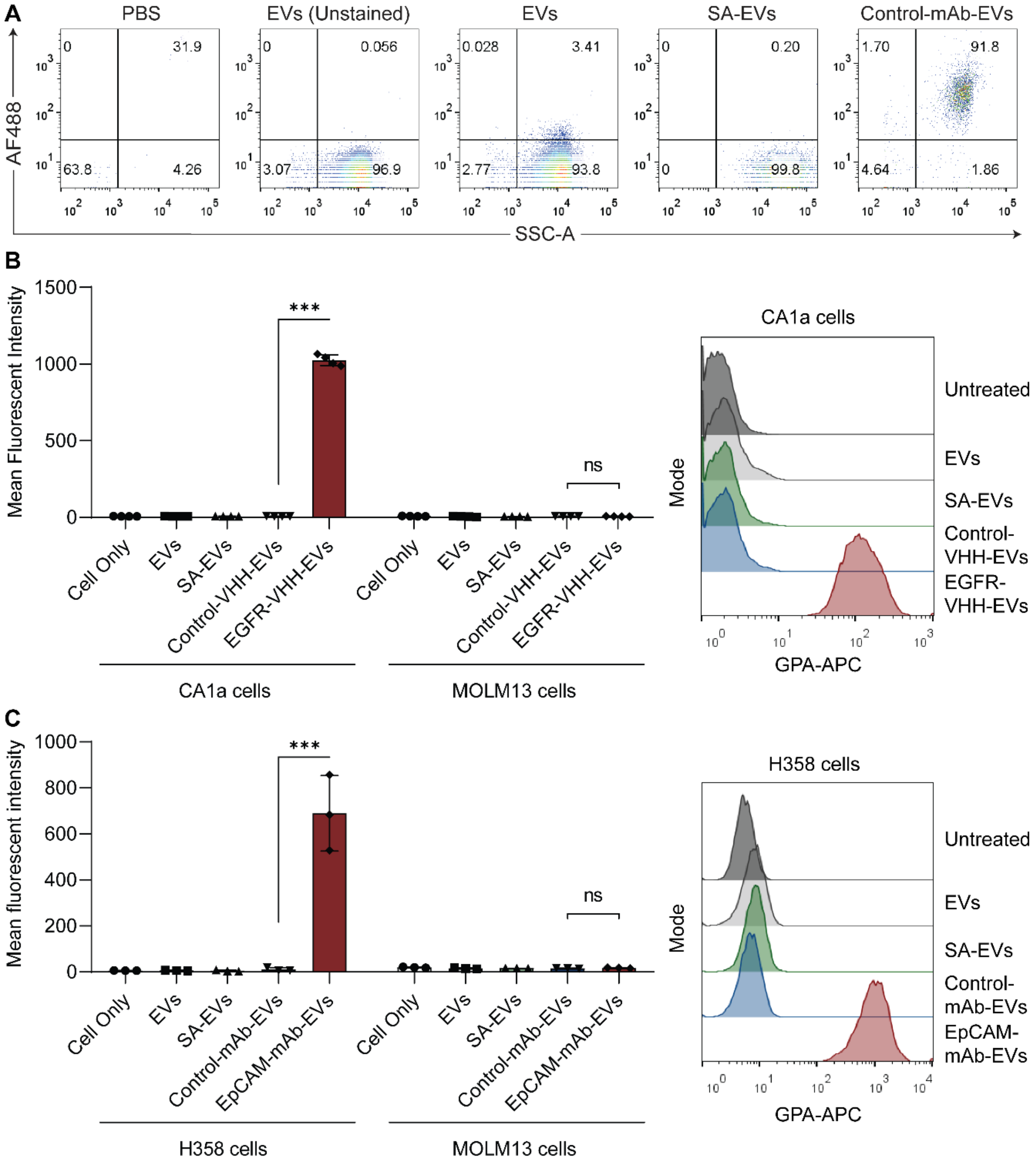

Figure S7| Antibody-conjugated EVs show increased affinity towards target cells. (A) Representative plots displaying the single EV flow cytometric analysis of monoclonal antibody conjugated EVs and control EVs following staining with an Alexa Fluor 488 conjugated secondary antibody. The plots display all events recorded within a fixed time period, with the events on the two quadrants on the right-side denoting events within the size range of EVs. **(B)** Binding of EGFR-VHH-conjugated RBCEVs and mCherry-VHH conjugated RBCEVs to EGFR-positive CA1a cells and EGFR-negative MOLM13 cells, presented as the mean intensity of GPA staining. **(C)** Affinity of EpCAM mAb or isotype control mAb-conjugated RBCEVs for EpCAM-positive H358 cells and EpCAM-negative MOLM13 cells, detected using an anti-GPA antibody conjugated to APC. Graphs in **A** and **B** represent mean ± SEM from 3 biological replicates. Student's one-tailed t-test: ns = not significant, ***P<0.001

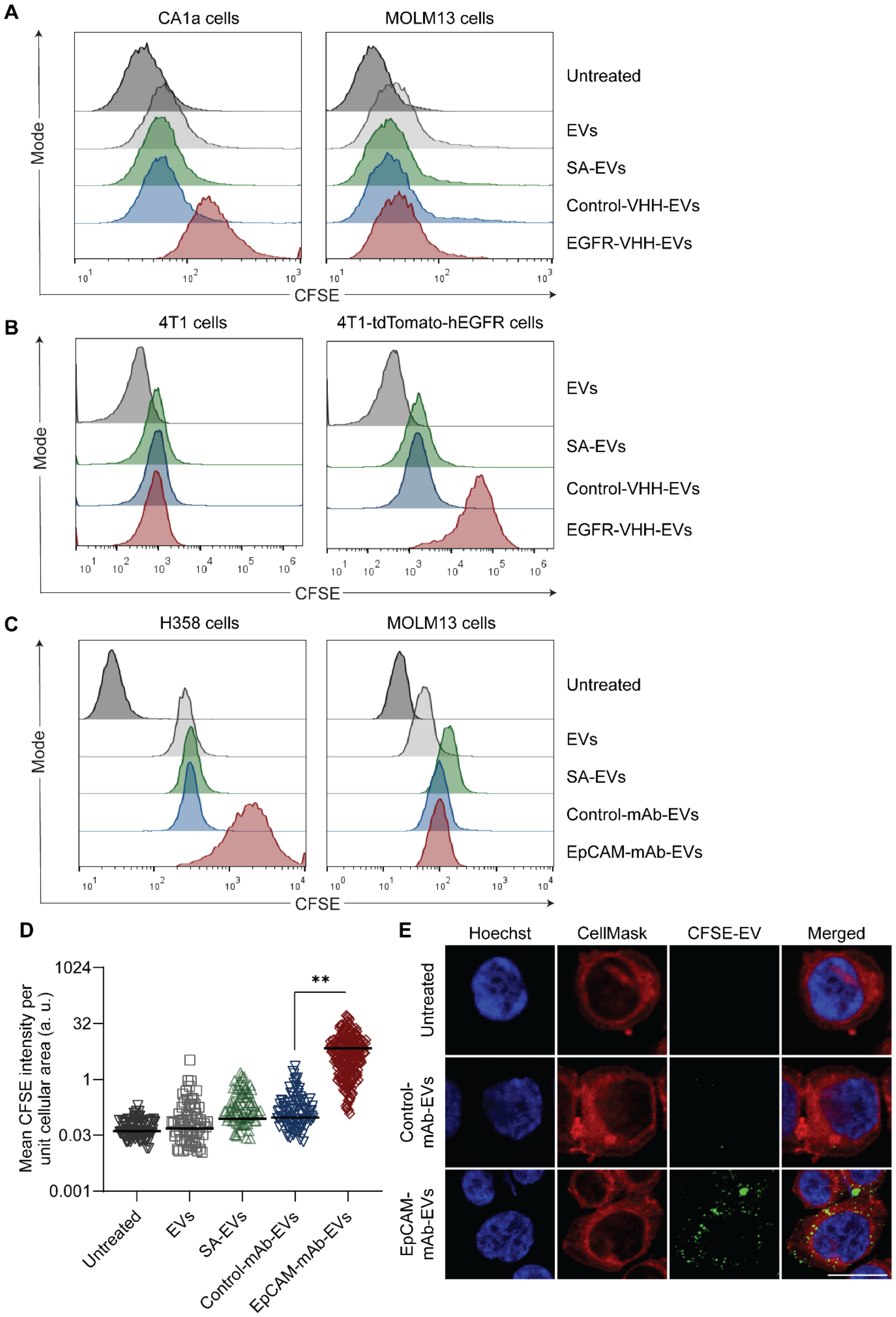

Figure S8| Uptake of antibody conjugated EVs by target cells. (A) Representative histograms displaying the CFSE fluorescence of CA1a and MOLM13 cells incubated with EGFR-targeting or control CFSE-labelled RBCEVs. **(B)** Representative histograms showing relative uptake of CFSE-labeled EVs by 4T1 and 4T1-tdTomato-hEGFR cells in a co-culture system. **(C)** Representative histograms displaying the CFSE fluorescence of H358 and MOLM13 cells incubated with EpCAM-

targeting or control CFSE-labelled RBCEVs **(D)** Mean CFSE fluorescence per unit cell area of H358 cells incubated with RBCEVs. The mean fluorescence from 256 cells chosen at random from each condition are presented in the graph. Student's one-tailed t-test: ** $P < 0.01$. **(E)** Z-stacks of H358 cells incubated for 2 hours with CFSE-labelled RBCEVs with or without EpCAM-mAb conjugation obtained using confocal microscopy. CFSE dye (green), CellMask (red), Hoechst (blue). Scale bar is 20 μm.

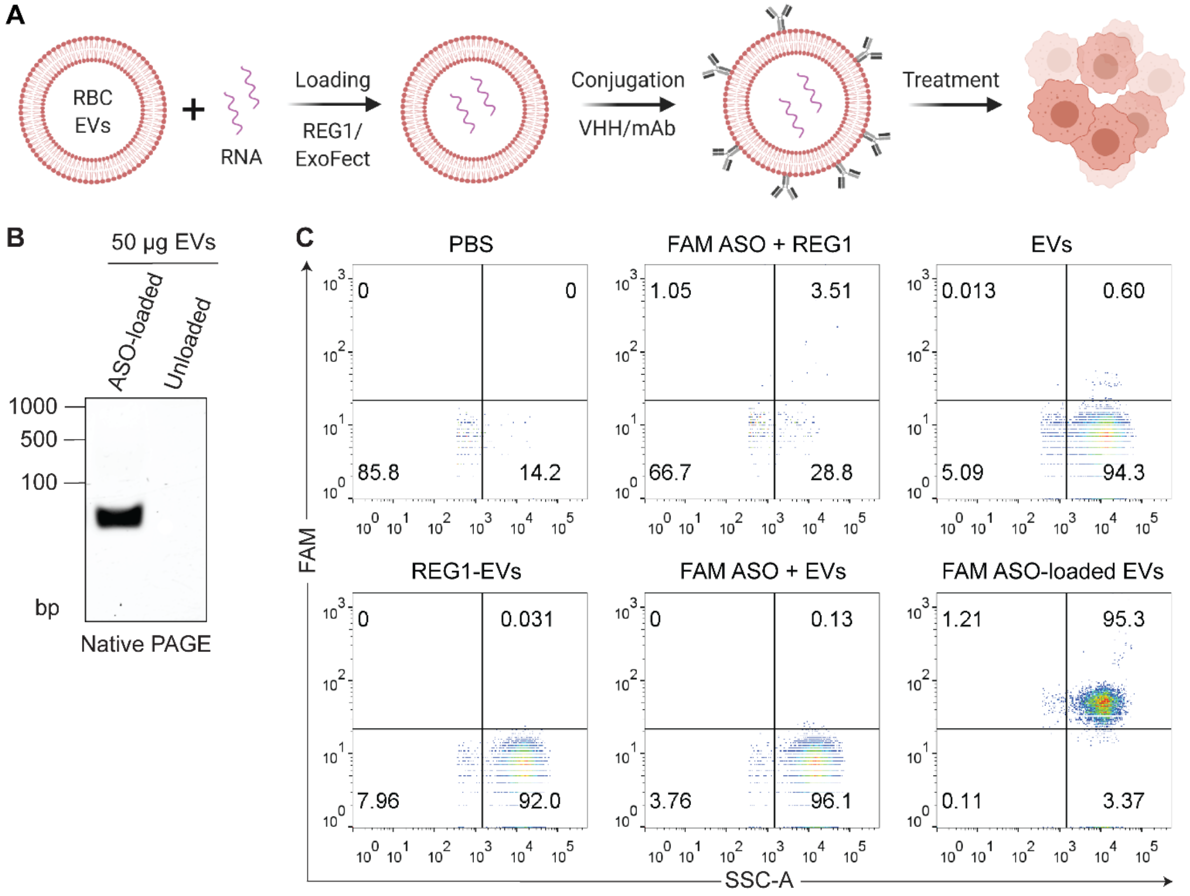

Figure S9| Characterization of RNA loading into RBCEVs. (A) Workflow for the preparation of engineered EVs for the targeted delivery of RNA payloads. RBCEVs were loaded with RNA cargoes followed by surface functionalization prior to incubation with cells. **(B)** Native PAGE analysis of ASO-loaded and unloaded RBCEVs, demonstrating the relative RNA content of exogenously loaded RNA vs endogenous RNA content. **(C)** Single EV flow cytometric analysis of FAM-conjugated ASO-loaded EVs. The plots display all events recorded within a fixed time period, with the events on the two quadrants on the right side denoting events within the size range of EVs. FAM fluorescence was acquired in the FITC channel.

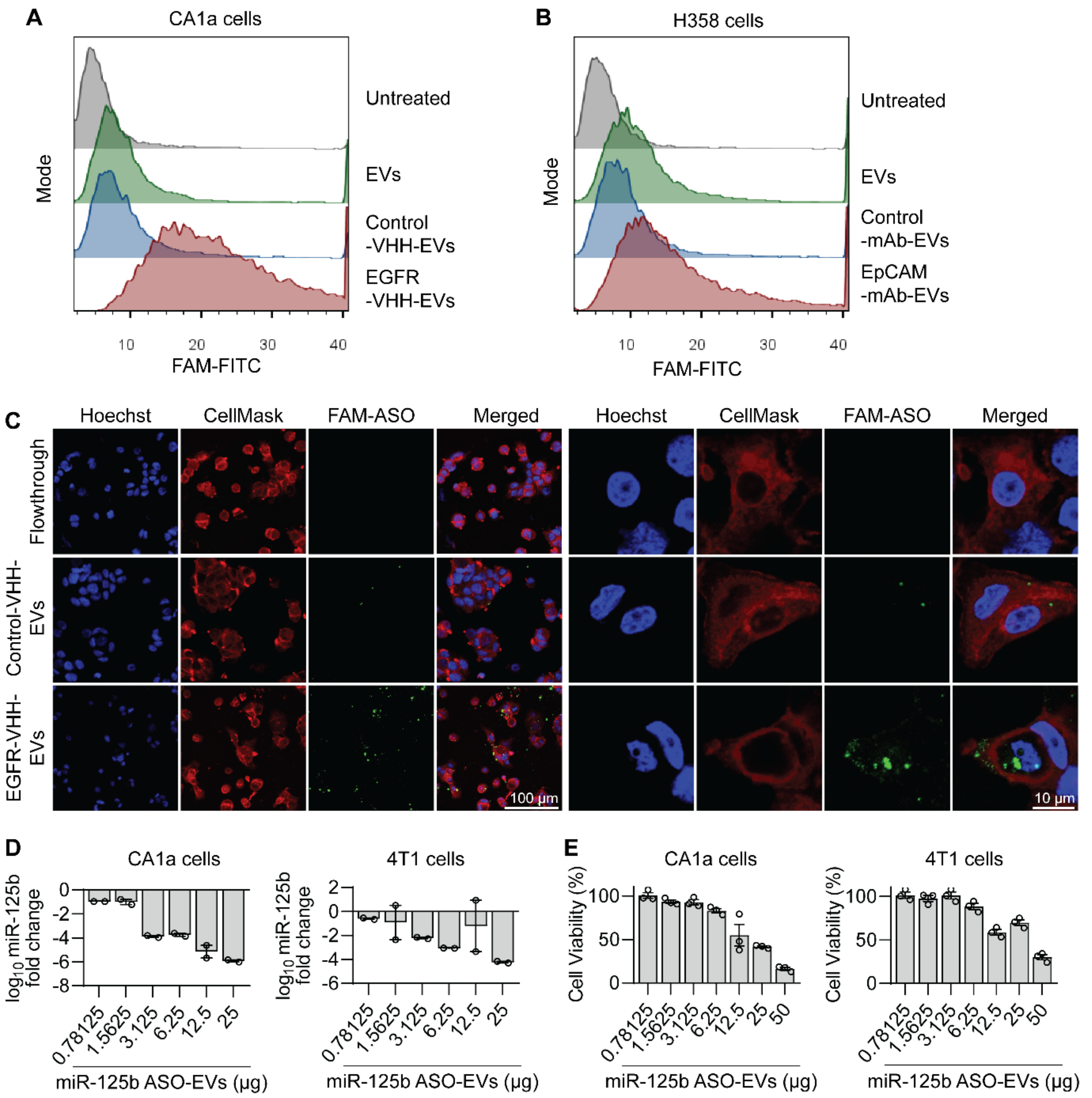

Figure S10| RNA delivery using antibody conjugated RBCEVs. (A-B) Representative histograms displaying the accumulation of FAM-ASO loaded EVs in CA1a cells **(A)** and H358 cells **(B)** with and without antibody conjugation. **(C)** Representative immunofluorescent images of CA1a cells incubated with EGFR targeting or non-targeting RBCEVs loaded with a FAM-conjugated ASO. Images were acquired at 20×. **(D)** Dose response for knockdown of miR-125b using increasing amounts of miR-125b ASO-loaded RBCEVs. Knockdown is presented as fold change in miR-125b expression normalized to U6B RNA (for CA1a cells) or snoRNA234 (for 4T1-tdTomato-hEGFR cells) relative to the untreated control. **(E)** Dose response curve assessing cell viability of CA1a and 4T1-tdTomato-hEGFR cells using CCK8 assay following treatment with increasing quantities of miR-125b ASO-loaded RBCEVs.

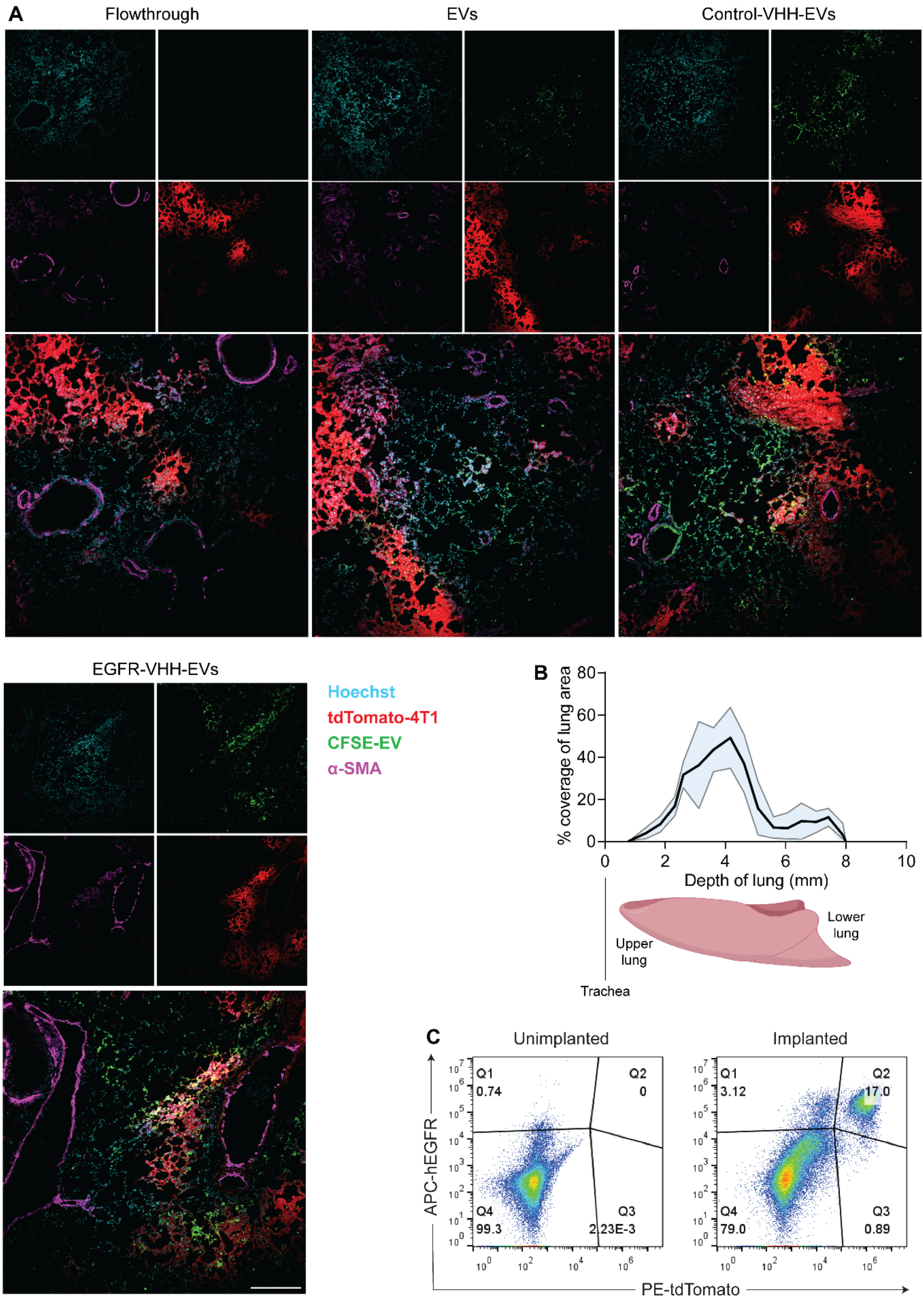

Figure S11| Distribution of intratracheally administered EVs. (A) Immunofluorescent imaging of lung sections from 4T1-tdTomato-hEGFR implanted mice administered intratracheally with a single dose of CFSE-labelled EVs with or without EGFR VHH conjugation. Sections were stained for α-SMA (magenta) and Hoechst (blue). EVs are visualized by the green punctate pattern while tumor tissue is visualized by the bright red tdTomato fluorescence. Scale bar, 200 µm. **(B)** Relative coverage of lung area with EVs obtained via immunofluorescent analysis of equally spaced transverse sections from an entire mouse lung (from top to bottom) administered with a single dose of CFSE-labelled EVs (n=3). Tile scans of transverse lung sections obtained approximately 500 µm apart from the top of the lung until the bottom were analyzed using ImageJ and the % of each section with EV signals was

quantified. Error bar (region in blue) represents the standard deviation across 3 sections at similar lung depth. **(C)** Representative flow cytometric gating of lung homogenates for separating out tumor cells from mouse lung cells for determining percentage of tumor cells remaining after treatment and for sorting out tumor cells from non-tumor cells for RT-qPCR analysis. Tumor cells were identified based on tdTomato expression and hEGFR surface staining with an hEGFR-APC antibody.

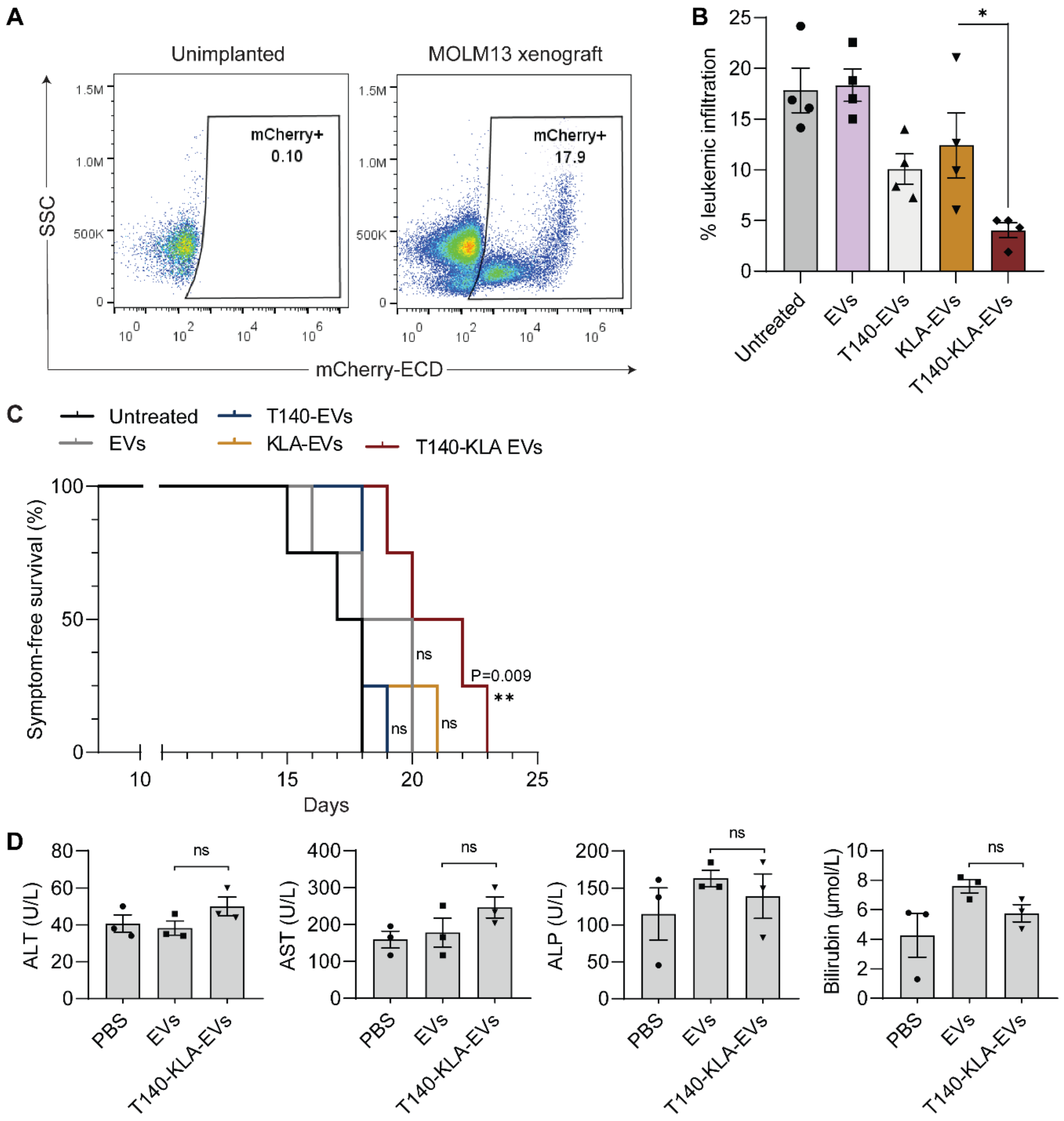

Figure S12| *In vivo* evaluation of T140-KLA-EVs for leukemia treatment. (A) Flow cytometric analysis of peripheral blood cells of unimplanted mice and mice implanted with MOLM13-Luc-mCherry cells. MOLM13 cells were gated out based on mCherry fluorescence acquired using the ECD channel. **(B)** Percentage of leukemic infiltration in the spleen for each treatment condition, represented as the percentage of leukemia cell nuclei observed by H&E staining. Data was obtained from 4 separate images acquired in a blinded manner. **(C)** Symptom-free survival rate of leukemic mice treated with unmodified EVs or T140, KLA or T140-KLA peptide coated EVs. Mantel-Cox test: **P < 0.01 (n = 4 mice). **(D)** ALT, AST, ALP and bilirubin levels in MOLM13 xenografted mice administered with a single dose of either uncoated EVs or T140-KLA-EVs (n = 3 mice). Graphs **B** and **D** display data as mean ± SEM. Student's one-tailed t-test: ns – not significant, * P < 0.05.

Table S1. List of peptides used in this study.

Peptide Name	Description	Amino acid sequence
TL5	Non-biotinylated control peptide	TRNGL
B-TL5	Linker peptide for streptavidin-mediated conjugation + detection and quantitation of ligation	Biotin-TRNGL
T140	CXCR4 targeting peptide for ligation	RRNalCYRKKPYRCitCR-GGGGS-NGL
B-T140	Biotinylated form of the T140 peptide for detection and quantitation	RRNalCYRKK(biotin)PYRCitCR-GGGGS-NGL
Scr-T140	Scrambled form of the T140 peptide for ligation	PCitKCRRRNalYRYKCR-GGGGS-NGL
Scr-B-T140	Biotinylated scrambled form of the T140 peptide for detection and quantitation	PCitK(biotin)CRRRNalYRYKCR-GGGGS-NGL
KLA	Pro-apoptotic peptide for ligation	KLAKLAKKLAKLAK- GGGGS-NGL
T140-KLA	Bifunctional apoptotic peptide	RRNalCYRK(-GG-KLAKLAKKLAKLAK)KPYRCitCR-GGGGS-NGL

T140 peptide: cysteine 4 and cysteine 13 are conjugated through a disulfide bridge; Nal: L-3-(2-naphthyl)-alanine; Cit: L-citrulline, B-T140: biotin conjugated to K 8, T140-KLA: KLA linked to K 8 with GG linker, Scr-T140: biotin conjugated to K 3.
